# High‐*T*_g_ Polythioesters From Isomerization Ring‐Opening Polymerization of a Renewable Oxanorbornane‐Fused Thionolactone

**DOI:** 10.1002/anie.6168208

**Published:** 2026-09-04

**Authors:** Eva Harsevoort, Henk‐Jan Akkerman, Martin Lutz, Arnaud Thevenon, Pieter C. A. Bruijnincx

**Affiliations:** ^1^ Organic Chemistry and Catalysis Institute for Sustainable and Circular Chemistry Faculty of Science Utrecht University Utrecht The Netherlands; ^2^ Structural Biochemistry Bijvoet Centre for Biomolecular Research Faculty of Science Utrecht University Utrecht The Netherlands

**Keywords:** biobased polymers, depolymerization, glass‐transition temperature, polythioesters, sustainable polymers

## Abstract

Polythioesters are gaining increasing interest due to their attractive material properties and improved end‐of‐life options. However, the lower ring strain of thiolactones compared to lactones limits the accessibility of polythioesters through conventional ring‐opening polymerization. Thionolactone isomerization ring‐opening polymerization provides an additional thermodynamic driving force, enabling polymer synthesis from more accessible monomers. Nevertheless, reported polythioesters typically exhibit low glass‐transition temperatures (*T*_g_), and the impact of sulfur substitution on the polymer(ization) properties remains poorly understood. Here, we report a biobased tricyclic thionolactone (**TCTn**) that undergoes selective polymerization to yield high‐molecular weight P(**TCT**) (ca. 100 kg mol^−1^). P(**TCT**) exhibits one of the highest *T*_g_ values reported for polythioesters (171°C), exceeding that of the analogous polyester (130°C). The thermally stable P(**TCT**) can be efficiently depolymerized to the unstrained thiolactone (**TCT**) through backbiting depolymerization. Whereas hydrolysis of P(**TCT**) under heterogeneous aqueous conditions is ten times slower than that of the polyester analogue, homogeneous methanolysis proceeds at comparable rates, indicating that the intrinsic susceptibility of the ester and thioester linkages toward nucleophilic attack is similar. Overall, this work provides insights into the design of sustainable, sulfur‐containing polymers, the intricacies of O→S substitution, and expands the space of high‐*T*_g_ norbornane‐fused polymers.

## Graphical abstract

A biobased tricyclic thionolactone undergoes selective isomerization ring‐opening polymerization to yield a high‐molecular‐weight polythioester with an exceptionally high *T*_g_ of 171°C, exceeding that of the analogous polyester. The polythioester efficiently depolymerizes through backbiting, while its hydrolysis and methanolysis reveal the critical influence of sulfur substitution and polymer end‐groups on degradation behavior.

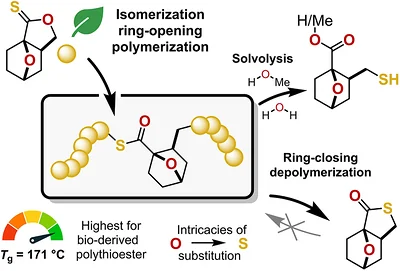

## 1 Introduction

The transition toward sustainable polymers, derived from renewable resources and designed with consideration for end‐of‐life, is essential to decrease greenhouse gas emissions and mitigate plastic pollution [1]. Ring‐opening polymerization (ROP) of (biobased) heterocyclic monomers has emerged as a promising route to renewable and recyclable polymers [2, 3, 4], particularly polyesters [5, 6] and polycarbonates [7, 8], and more recently also their sulfur analogues [9], polythio(no)esters [10, 11] and polythiocarbonates [12, 13]. Biobased polymers are often rich in oxygen‐containing functional groups, while examples of polymers carrying nitrogen, sulfur, or phosphorus functional groups are less abundant. Heteroelement replacement, for example, by swapping oxygen atoms for sulfur, can expand the application space of these polymers substantially [14]. Sulfur‐containing polymers in general [12, 15, 16, 17, 18, 19, 20, 21, 22, 23, 24] and polythioesters in particular [25, 26, 27, 28, 29, 30] are of great interest due to advantages in material properties such as enhanced thermostability [31], crystallinity [32, 33, 34], potential for dynamic bonding [11, 35, 36, 37, 38] and mechanical performance [39]. However, they also generally exhibit a lower glass‐transition temperature (*T*_g_), which limits their use in applications requiring stiffness at elevated temperatures. With regard to end‐of‐life, polythioesters are reported to exhibit improved recyclability [38, 40] and higher degradability [16, 41] than polyesters owing to the higher lability of the thioester bond [10, 42]. Although polythioesters are typically more stable than polyesters under acidic and neutral conditions, under alkaline conditions their solvolysis is usually faster [28]. Such improved end‐of‐life properties have been reported, for example, for poly(*rac*‐thiolactide) versus poly(*rac*‐lactide) [42]. Nevertheless, direct comparative studies of structurally analogous poly(thio)esters remain scarce.

Polymers formed by ROP are generally more amenable to chemical recycling to the monomer under milder conditions than those produced by highly exergonic polymerization methods (e.g., olefin polymerization), since ROP of cyclic monomers is in equilibrium with the ring‐closing depolymerization (RCD) reaction. Understanding and controlling this equilibrium is a key challenge in the design of polymers that can be synthesized in high conversion under convenient conditions, while remaining readily recyclable to their monomers. This balance is dictated by the ceiling temperature (*T*_c_), defined as *T*_c_ = Δ*H*_p_/(Δ*S*_p_° + R ln[M]_eq_) [43, 44]. Thiolactones generally display lower *T*_c_ values than the analogous lactones. For example, *T*_c_ decreases markedly from lactide to monothiolactide to thiolactide (*T*_c_ = 284°C, 106°C, −67°C at 1 M, resp.) [26, 40]. A similar trend is observed for δ‐valerolactone and thio‐δ‐valerolactone (*T*_c_ = 155°C and −66°C, resp.) [45, 46]. Furthermore, ROP of γ‐thiobutyrolactone (TGBL), the thio‐equivalent of the low‐strain lactone γ‐butyrolactone (GBL, *T*_c_ = −136°C at 1 M) [47] does not occur (Scheme 1B) [48]. These trends highlight that the reduced ring strain in thiolactones disfavors ROP, underscoring the need for sufficient thermodynamic driving force for efficient polymerization. A strategy to increase the thermodynamic driving force for ROP is the use of fused [49, 50, 51] or bridged [52, 53, 54] multicyclic monomers which provide more negative enthalpies of ring‐opening (Δ*H*_p_). The bridged bicyclic thiolactone ^[221]^BTL exhibits a more balanced ring strain, resulting in a system with efficient ROP while still allowing for complete depolymerization back to the monomer (*T*_c_ = −20°C at 1.0 M) [33]. However, the synthesis of this monomer (four steps from cyclopentene carboxylic acid) illustrates the often‐cumbersome routes to thiolactones. In general, they are prepared via cyclization of mercaptocarboxylic acids, which themselves are not readily available from biomass [10]. Indeed, thiolactones that are readily accessible from biomass [55] (e.g., homocysteine thiolactone) [56] are typically five‐membered and unstrained. The synthesis of suitably strained analogues is thus not straightforward [10]. Overall, polythioesters are highly attractive materials compared to their polyester counterparts, but the synthetic accessibility of appropriate thiolactones significantly limits the available monomer space for ROP [57].

**SCHEME 1:**
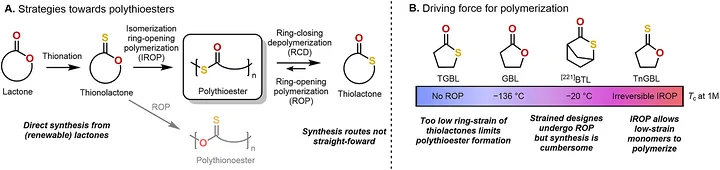
(A) Isomerization ring‐opening polymerization (IROP) strategy for polythioester synthesis and (B) the enthalpy gain from isomerization allows for low‐strain monomers to undergo polymerization without the need for strained monomer design.

In stark contrast, isomerization ring‐opening polymerization (IROP) of thionolactones to form polythioesters overcomes both the low thermodynamic driving force and the limited accessibility of suitable monomers, as thionolactones can be synthesized directly through thionation of available lactones [58, 59]. IROP exploits the enthalpy gain from the thiocarbonyl to carbonyl conversion [(C═S)─O to (C═O)─S], allowing even low‐strain monomers to undergo polymerization. For example, when GBL is thionated to γ‐thionobutyrolactone (TnGBL), IROP to the corresponding polythioester P(TGBL) occurs at temperatures up to 80°C, as first reported by Endo et al. [48] and later improved by Hong et al. [27] (Scheme 1B). Next to TnGBL, IROP of other thionolactones has been reported by the groups of Hong [60, 61] Gutekunst [57] and Chen [62] (Figure 1A). Although IROP is thermodynamically more favorable, it may still have to compete with the ROP pathway (i.e., polymerization without isomerization), resulting in the formation of polythionoester linkages [63, 64, 65]. Full selectivity for IROP can be achieved through appropriate choice of initiator/catalyst system, for example, a mild tetrabutylammonium thioacetate initiator [57]. Thionolactone IROP then generally proceeds rather cleanly with only minor amounts of isomerized thiolactone formed during polymerization. Monomers containing acidic α‐hydrogens, however, do give rise to dimeric side products if basic IROP conditions are used [27].

**FIGURE 1:**
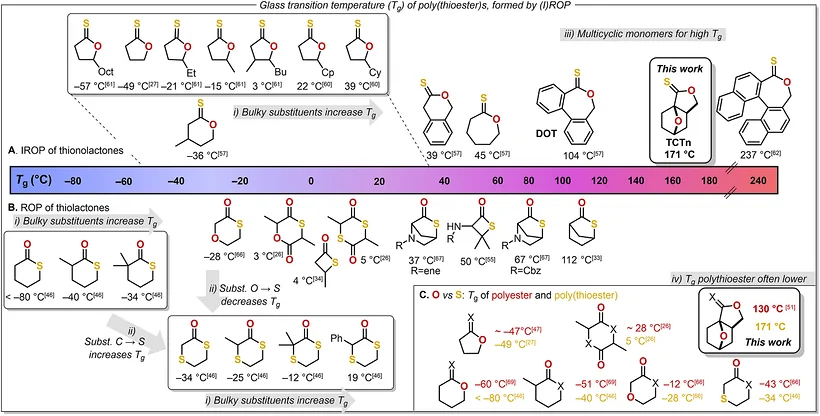
Trends observed in the glass‐transition temperature (*T*_g_) of a selection of reported polythioesters. (A) Polythioesters from thionolactone IROP and (B) from thiolactone ROP; **C**) Comparison of polythioesters with analogous polyesters. Labels *i*–*iv* denote the four design trends discussed in the text.

Polythioesters, whether formed via IROP of thionolactones (Figure 1A) or ROP of thiolactones (Figure 1B), typically exhibit low glass‐transition temperatures (*T*_g_). Several monomer design strategies have been explored to increase *T*_g_. First, substitution patterns directly on the thio(no)lactone ring can tune *T*_g_ by restricting rotation of the polymer backbone (Figure 1, *i*) [40]. This effect is observed for different monomer series; substituted γ‐thionolactones showed *T*_g_ values ranging from −57°C (long alkyl substituent) [61] to 39°C (bulky cyclohexyl group) [60], for substituted δ‐thiolactones, the *T*_g_ increased from below −80°C for poly(δ‐thiovalerolactone) to −34°C upon dimethyl substitution, while for 1,4‐dithianone‐based polythioesters the *T*_g_ increased from −34°C to 19°C with phenyl substitution. A second strategy is to incorporate heteroatoms into the monomer to influence *T*_g_, (Figure 1, *ii*), although structure‐property relationships remain insufficiently understood for these systems [12]. For the δ‐thiolactones, sulfur incorporation (C→S) was reported to increase *T*_g_ [46] whereas for thioester‐ethers it was observed that exchanging ethers for thioethers (O→S) instead lowered the *T*_g_ [66]. A third approach (Figure 1, *iii*) involves the use of rigid multicyclic thio(no)lactones to yield polythioesters with higher *T*_g_ values than their monocyclic analogues, as demonstrated for bridged bicyclic thiolactones (up to 112°C) [33, 67] and fused thionolactones [57, 62]. More generally, most reported polythioesters exhibit a lower *T*_g_ than the analogous polyester, as illustrated by the lactide → monothiolactide → dithiolactide series (Figure 1, *iv*); this has been attributed to the higher chain mobility due to the lower partial double‐bond character in thioesters [12, 26, 68, 69]. In summary, trends in reported glass transition temperatures show that, for polythioesters, *i*) bulky substituents increase *T*_g_, *ii*) the effect of replacing a backbone atom with sulfur depends on whether an O or C atom is replaced, *iii*) rigid multicyclic monomers lead to higher *T*_g_ values, and *iv*) polythioesters typically exhibit lower *T*_g_ values than their polyester analogues.

To date, the highest reported *T*_g_ value for a polythioester is, to the best of our knowledge, 237°C, formed from IROP of a bis(naphthalene)‐fused thionolactone (Figure 1A) [62]; however this monomer is not readily available from biomass. Examples of high *T*_g_ polythioesters derived from renewable monomers remain very limited.

Building on our previous work on high *T*_g_ polyesters from ROP of bio‐based tricyclic lactones (**TCL**) [51], we herein report on the reactivity of the related thionolactone (**TCTn**). IROP of **TCTn** affords the high molecular weight (MW) polythioester P(**TCT**), which exhibits one of the highest reported *T*_g_ (171°C) for polymers prepared by ROP or IROP. We demonstrate its ability to undergo RCD, as well as methanolysis and hydrolysis, and directly compare these depolymerization pathways with those of its direct polyester analogue. Presenting a rare direct comparison between polyester and polythioester systems, this study highlights the critical impact of O→S substitution on end‐of‐life behavior as well as on the materials’ thermal properties, thereby providing insights for the development of sustainable polymer materials.

## 2 Results and Discussion

### 2.1 Monomer Synthesis

Thionation of **TCL** was performed under modified literature conditions [70] using Lawesson's reagent (0.8 equiv.) at 150°C for 21 h, cleanly affording **TCTn** with 80% conversion, which was isolated in 67% yield after column chromatography (Scheme 2). **TCTn** absorbs weakly in the violet region (λ_max_ of 399 nm; ε = 18.8 L mol^−1^ cm^−1^, Figure 2D). This weak absorbance is the result of the forbidden n→π* transition of the thiocarbonyl group [71] and is comparable to ε‐caprothionolactone (λ_max_ = 388 nm, ε = 17 L mol^−1^ cm^−1^) [72].

**SCHEME 2:**
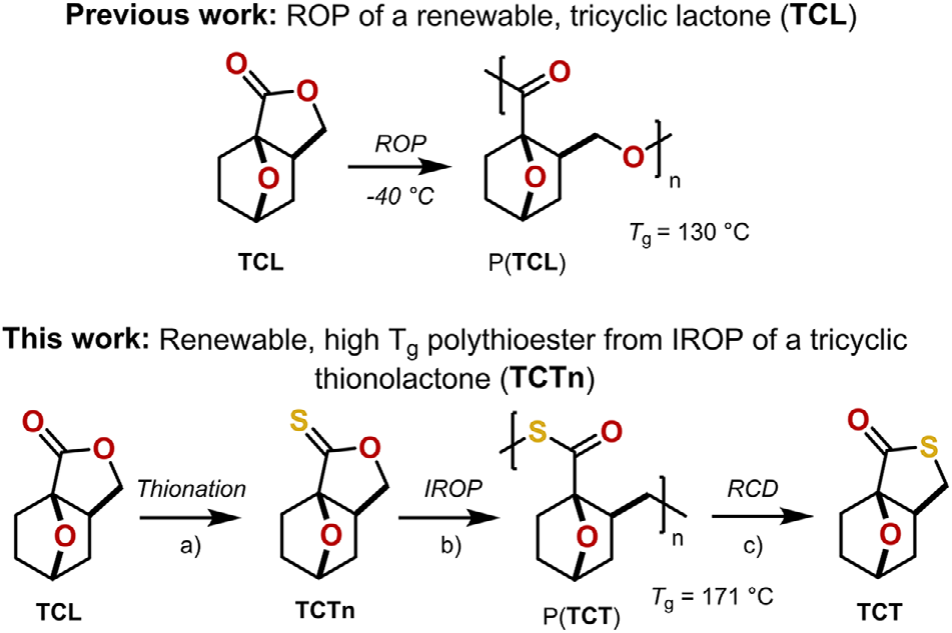
Overview of tricyclic lactone ROP and analogous IROP of tricyclic thionolactone **TCTn** and subsequent ring‐closing depolymerization (RCD) to the tricyclic thiolactone (**TCT**). Conditions: a) Lawesson's reagent (LR, 0.8 equiv.), toluene, 150°C 21 h, 67% isol. yield; b) ^t^Bu‐P_4_/BnOH in toluene or NBu_4_SAc in *N,N*‐dimethylacetamide, 80°C; c) TBD (0.5 equiv.), DCM, 20°C, 24 h, 77% isol. yield.

**FIGURE 2:**
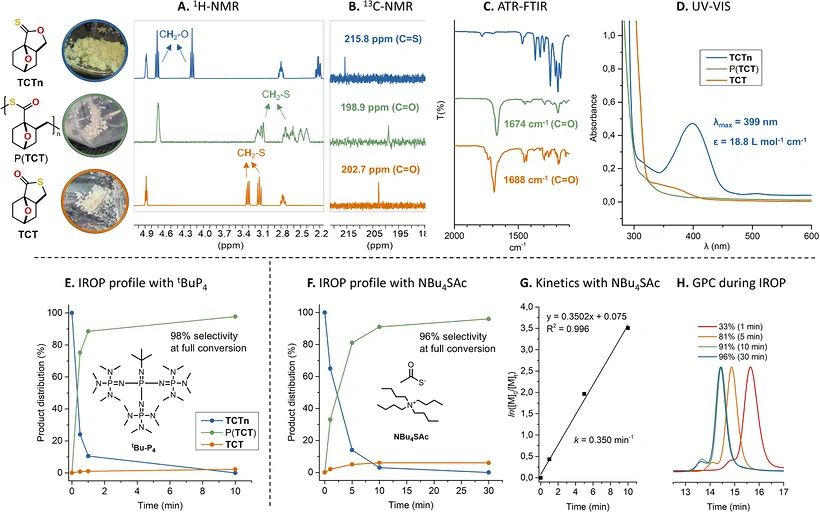
(A) ^1^H‐NMR and (B) ^13^C‐NMR spectra (CDCl_3_) with the typical C=S and C=O chemical shifts, (C) ATR‐FTIR showing the C═O stretch vibration after isomerization, and (D) UV‐VIS spectra of the thionolactone **TCTn**, polythioester P(**TCT**) and thiolactone (**TCT**) in DCM (25 mM); (E) Isomerization ring‐opening polymerization (IROP) profile of **TCTn**, catalyzed by ^t^Bu‐P_4_ and (F) catalyzed by NBu_4_SAc; (G) semilogarithmic kinetic plot and (H) overlay of the normalized GPC traces at various monomer conversions.

### 2.2 Polymer Synthesis and Characterization

IROP of thionolactone **TCTn** was explored with polyaminophosphazene superbase ^t^Bu‐P_4_ with benzyl alcohol as initiator [27], as well as tetrabutylammonium thioacetate (NBu_4_SAc) [57]. Both ^t^Bu‐P_4_ and NBu_4_SAc have been reported to promote rapid and selective IROP of thionolactones. Excitingly, both catalysts/initiator systems afforded full **TCTn** conversion and formation of the polythioester P(**TCT**), as observed from ^1^H‐NMR analysis by the lower chemical shift of the methylene resonance (Figure 2A). Complete isomerization to the polythioester P(**TCT**) was further confirmed by the ^13^C‐NMR spectrum of the isolated polymer, showing only the characteristic carbonyl (C═O) chemical shift at 199 ppm and no thiocarbonyl signal (C═S), originally found at 216 ppm for **TCTn** (Figure 2B). Infrared (IR) analysis also showed a C═O stretch vibration for P(**TCT**) (Figure 2C). Consistently, the yellow color disappeared during polymerization and P(**TCT**) is obtained as a white powder (Figure 2) after precipitation from methanol. The full isomerization to polythioester confirms a S_N_2‐type mechanism where the thiocarboxylate anion acts as the propagation species (see Figure S1), as typically observed for weaker nucleophiles [10, 73].

The ^t^Bu‐P_4_‐catalysed IROP proceeded rapidly, reaching full conversion within 10 min at 80°C, whereas under the same conditions the NBu_4_SAc‐initiated reaction was slower, completing in 30 min (Figure 2E,F, respectively). The semilogarithmic kinetic plot (at 100:1 [**TCTn**]:[NBu_4_SAc] loading, Figure 2G) shows first‐order kinetics in the monomer, with an observed rate constant of 0.35 min^−1^. This is more than an order of magnitude faster than the IROP of thionovalerolactone under identical conditions (*k*_obs_ = 0.0218 min^−1^) [57].

The polythioester P(**TCT**) formed with high selectivity at full conversion, with both ^t^Bu‐P_4_ (98% sel.) and NBu_4_SAc (96% sel.) as catalyst (Figure 2E,F). Side products from Claisen‐like condensation reactions are prevented in the case of **TCTn**, given its lack of α‐hydrogens adjacent to the thiocarbonyl. The only side product that could be observed in minor amounts is the isomerized, ring‐closed thiolactone **TCT**, formed by backbiting during polymerization [27, 61]. This was further confirmed by independent synthesis of the thiolactone isomer **TCT** directly from the lactone **TCL** using potassium thioacetate [74]. It is worth noting that in the direct synthesis of **TCT**, it could only be isolated in very low yield of 5%, which again underscores the challenges to prepare thiolactones from lactones, compared to thionolactones. A series of experiments showed that extending the reaction time increased the formation of **TCT** as the thermodynamic product to 7% after 2 h. After 21 h under polymerization conditions, 25% of the polymer was depolymerized to **TCT** via backbiting and after 46 h, 91% of the polymer had converted (Figure S2 and Table S2). These results indicate that consecutive RCD is thermodynamically favored (see discussion below) and that paying careful attention to the reaction time is crucial to achieve high selectivity toward P(**TCT**).

Both catalysts yield P(**TCT**) with expected MWs and moderately narrow dispersity (Đ = 1.1–1.4, Table 1). Surprisingly, polythioester prepared with either catalyst consistently showed a high‐MW shoulder in the gel permeation chromatography (GPC) trace at approximately twice the main polymer MW (Figures 2H and 3B). This double‐MW species appeared as early as 30 s into the polymerization with ^t^Bu‐P_4_, increasing to 11% of the GPC area after 1 min and to 32% after 10 min (Table S2). With NBu_4_SAc, a smaller fraction of double‐MW polymer was observed (8% after 30 min, Figure 2H and Table S3). The double‐MW shoulder became more pronounced when higher MW polymers were targeted (Figure 3B). Notably, the fraction of the double‐MW species did not increase upon long‐term storage of the isolated polymers (Figures S74 and S98). Similar bimodality has previously been reported for thionolactone IROP and attributed to coupling between thiocarboxylic acid chain‐ends [61]. Addition of the reducing agent dithiothreitol (DTT) to a P(**TCT**) sample, anticipated to cleave the S─S bonds in the double‐MW polymers, led to a decrease in the double‐MW peak and an increase in the main, single‐chain distribution (Figure 3C), consistent with the assignment of the shoulder to coupled polymer chains.

**TABLE 1:** Isomerization ring‐opening polymerization of thionolactone **TCTn** ^a^.

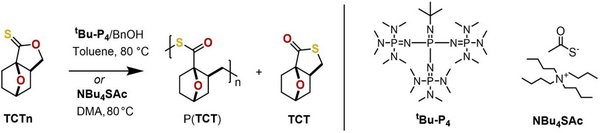									
Entry	Cat/Init.	Ratio [**TCTn**]/[Cat/Init.]^b^	Time	Sel.^c^ (%)	Yield (%)	*M*_n, calc_ (kg mol^−1^)^d^	*M*_n, NMR_ (kg mol^−1^)^e^	*M*_n, GPC_ (kg mol^−1^)^f^	Đ^g^ (% high MW chains)
1	^t^Bu‐P_4_/BnOH	100/1	10 min	98	78	16.8	—	25.0	1.22 (37.7)
2	NBu_4_SAc	100/1	1 h	96	54	16.4	22.2	21.7	1.08 (8.0)
3	NBu_4_SAc	200/1	1 h	94	79	32.1	36.0	37.6	1.17 (23.9)
4	NBu_4_SAc	400/1	2 h^h^	94	40	64.1	69.7	64.2	1.31 (38.8)
5	NBu_4_SAc	600/1	3 h	95	87	97.1	103.2	75.3	1.34 (36.6)
6	NBu_4_SAc	800/1	3 h	94	73	128.1	131.5	91.9	1.39 (48.0)

^a^ Polymerization conditions: 80°C [**TCTn**]_0_ = 1 M (toluene for ^t^Bu‐P_4_/BnOH, *N,N*‐dimethylacetamide for NBu_4_SAc). Conversion of **TCTn** was >99% for all entries.

^b^ For entry 1, ^t^Bu‐P_4_ and BnOH were used in a 1/1 ratio, so [**TCTn**]/[Cat.] = [**TCTn**]/[Init.] = 100/1. For entries 2–6 the ratio is [**TCTn**]/[NBu_4_SAc], which acts as the initiator.

^c^ Selectivity (Sel.) determined by ^1^H‐NMR analysis of the relative integrals of P(**TCT**) and the only observed side product, **TCT**.

^d^ *M*_n, calc_ calculated as conversion × selectivity × [**TCTn**]/[Init.] × MW of monomer + MW of initiator.

^e^ *M*_n, NMR_ as determined from chain length n based on end‐group analysis × MW monomer + MW initiator.

^f^ M_n, GPC_ determined by gel permeation chromatography at 30°C in chloroform, relative to polystyrene standards.

^g^ Đ = *M*_w, GPC_/*M*_n, GPC_. All samples exhibit bimodal MW distributions; the percentage of high MW signal from the GPC integral is depicted in brackets. The reported *M*_n, GPC_ and Đ were calculated from the combined integration of both peaks. Data from separately integrated signals are provided in Figures S61, S73, S82, S89, S97, and S105.

^h^ 98% conversion of **TCTn** was measured after 1 h, full conversion was reached after 2 h.

**FIGURE 3:**
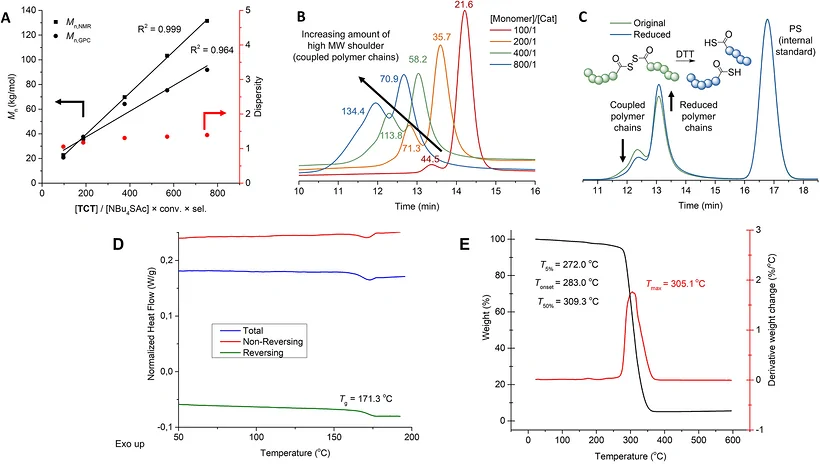
(A) Plot of *M*_n, NMR_ and *M*_n, GPC_ and the dispersity of polythioester P(**TCT**) as a function of the NBu_4_SAc catalyst loading, (B) overlay of the GPC traces of the polymerization with different catalyst loadings with M_p_ indicated in kg mol^−1^ (600/1 omitted for clarity) and (C) reduction with dithiothreitol (DTT) resulted in a decreased double‐MW signal compared to the polystyrene (PS) internal standard. (D) MDSC thermogram (second heating scan) and (E) thermogravimetric analysis (TGA, black) and derivative thermogravimetry (DTG, red) curve of P(**TCT**) from Table 1, entry 6 (ratio **TCTn**/NBu_4_SAc 800/1).

For the synthesis of high MW polymers we resorted to the NBu_4_SAc catalyst, which is slower but does not suffer from the catalyst/polymer separation issues encountered with ^t^Bu‐P_4_, and affords polymers with improved thermal stability without requiring extensive purification (see Supporting Information). Good control of the molar mass of P(**TCT**) was achieved when varying the ratio of [**TCTn**]:[NBu_4_SAc], and high MW polymers (>100 kDa) were formed within 3 h with 0.125 mol% catalyst, while maintaining full conversion of **TCTn**. The number average MW determined from the thioacetate end‐group analysis from ^1^H‐NMR (*M*_n, NMR_) very closely matches the theoretical values (*M*_n, calc_) across the different catalyst loadings (Table 1, entries 2–5 and Figure 3A). This strong correlation also indicates high end‐group fidelity. The MWs measured with GPC (*M*_n, GPC_) calibrated against polystyrene showed a similar trend, but deviate more from *M*_n, calc_ at lower catalyst loadings. This may arise from differences in the solution conformation (coiling) of higher‐molecular‐weight polythioesters, leading to changes in hydrodynamic volume. Alternatively, the shift in end‐group composition with increasing MW (from thiocarboxylic acid‐terminated chains toward a greater fraction of disulfide‐coupled chains) may influence polymer‐column interactions and affect the measured elution time. Despite the shoulder attributed to the double‐MW chains, the GPC traces indicated narrow dispersity (Đ = 1.1–1.4), lower than previously reported for polythioesters synthesized with the same catalysts (Đ = 1.2–2.2 for NBu_4_SAc [57] and 1.5–1.7 for ^t^Bu‐P_4_ [27]).

### 2.3 Thermal Properties

The thermal properties of polythioester P(**TCT**) were investigated with modulated differential scanning calorimetry (MDSC) and thermogravimetric analysis (TGA). MDSC measurements indicate that polythioester P(**TCT**) is amorphous, exhibiting a glass‐transition temperature (*T*_g_) around 170°C, and no melting events. The *T*_g_ occurs at 167°C for polymers formed with a ratio **TCTn**/NBu_4_SAc of 100/1 (*M*_n, NMR_ = 22.2 kg/mol, Table 1, entry 2) and this value slightly increases with MW before plateauing at 171°C (Figure 3D and Table S1). This *T*_g_ is among the highest reported for polythioesters formed by (I)ROP, and the highest for non‐aromatic polymers, only surpassed by the polythioester from a bis(naphthalene)‐fused thionolactone (*T*_g_ = 237°C, Figure 1A) [62]. Remarkably, P(**TCT**) exhibits a *T*_g_ ca. 40°C higher than the analogous polyester P(**TCL**) (*T*_g_ = 129.6°C) [51]. As discussed above, most polythioesters show *T*_g_ values that are lower than their polyester analogues. The P(**TCL**) versus P(**TCT**) couple deviates from this general trend, highlighting again that O→S substitution does not constitute a universal design rule for *T*_g_ modulation and can give rise to non‐intuitive structure‐property relationships whose origins remain to be fully elucidated. The decomposition temperature at 5% weight loss (*T*_5%_) was determined to be 272.0°C (Figure 3E), consistent with other polythioesters [46, 57] and close to the analogous polyester (*T*_5%_ = 261.2°C).

### 2.4 Thermodynamic Analysis of Isomerization and Polymerization

Full IROP conversion of **TCTn** to polythioester P(**TCT**) indicates that, as expected, the isomerization is energetically favorable, while the observed consecutive RCD after extended polymerization times suggests that the closed thiolactone **TCT** is the thermodynamically most stable species. To evaluate the relative stabilities of the thionolactone, polythioester and thiolactone, ground state energies were computed using density functional theory (DFT) (Figure 4A). P(**TCT**) was approximated as a single repeat unit capped with two thioester groups, by virtually ring‐opening the monomeric structure with methyl thioacetate. IROP of **TCTn** was calculated to be exergonic (ΔG_298K_ = −12.3 kcal mol^−1^), and ROP of **TCT** was computed to be endergonic (4.1 kcal mol^−1^). These results suggest that ROP of **TCT** is inaccessible. Indeed, attempts at polymerization of **TCT** at low temperatures (−40°C) were unsuccessful. In addition, these calculations predict favorable RCD of the polythioester to **TCT** as is also experimentally observed. Furthermore, ring‐opening of the thionolactone **TCTn** to the polythionoester repeat unit (without isomerization) is also calculated to be endergonic (ΔG_298K_ = +2.4 kcal mol^−1^), in line with the observed full selectivity for IROP versus ROP (i.e., no mixed backbone, complete isomerization observed).

**FIGURE 4:**
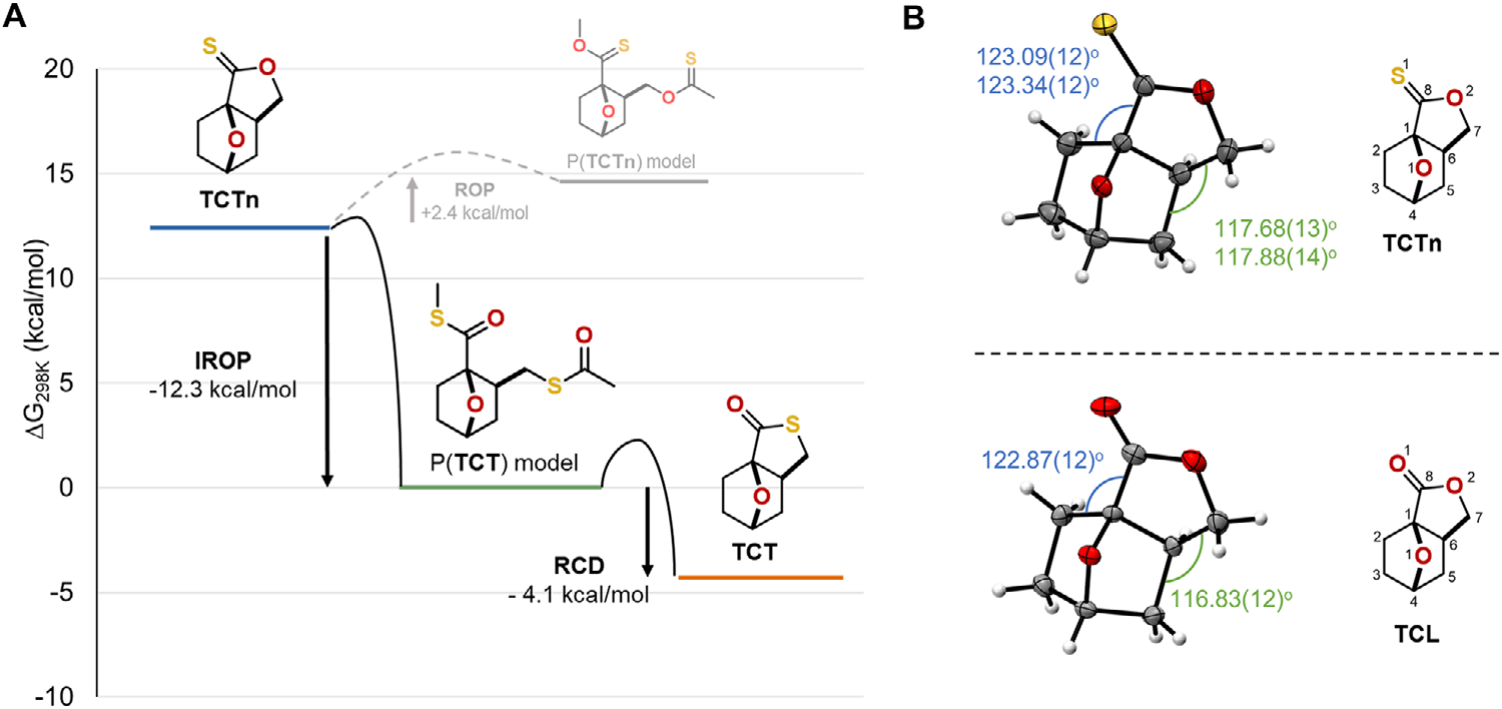
(A) Free energies computed with DFT (B3LYP at 6‐311+g(d,p) and gd3bj dispersion in vacuum) of **TCTn**, **TCT** and the capped polythio(no)esters repeat units as model P(**TCT**) and P(**TCTn**). The energies were normalized using the energies of methyl thioacetate (to polythioester) or methyl thionoacetate (to polythionoester), see Figure S19. (B) Solid state structure of **TCTn** and **TCL** [51]. Ellipsoids are drawn at 50% probability, and relevant angles are highlighted in green and blue, indicating the relatively low angular strain. Only one of the two independent molecules of **TCTn** in the asymmetric unit is depicted.

To probe the (lack of) strain in **TCTn**, we determined its geometric parameters by single crystal x‐ray diffraction (Figure 4B). Two independent **TCTn** molecules are present in the asymmetric unit, with very similar geometries (r.m.s. deviation = 0.03 Å). The structure of **TCTn** was found to be highly similar to lactone **TCL** [51]; the largest bond length difference between the two structures is 0.021(3)–0.022(3) Å (C8−O2), and the largest bond angle difference is 0.24(18)°–0.54(18)° (C1−C8−O2). Previously, we found that strain in these multicyclic monomers is better reflected by the angles around the bridgehead than by the structural parameters of the five‐membered lactone ring [51, 75]. The variation in these angles between **TCTn** and **TCL** (highlighted in blue and green in Figure 4B) is less than 1°, suggesting that the ring strain in **TCTn** is similar to that of **TCL** (*T*_c_ of −21°C at 1 M). This observation aligns with the endergonic ROP calculated with DFT, and that polymerization can only be achieved with the additional enthalpic contribution of (C═S)─O isomerization. Indeed, whereas **TCTn** can be polymerized to the polythioester using isomerization as driving force, lactone **TCL** does not have this option and did not react under the same conditions (Table 1, entry 1), in line with its low *T*_c_ [51].

### 2.5 Ring‐Closing Depolymerization

Encouraged by the observed RCD of P(**TCT**) from backbiting during IROP after prolonged reaction times, and as also seen at high temperatures in TGA/DSC, depolymerization was investigated under solution‐phase conditions with organic bases known to promote polythioester RCD [10]. With ^t^Bu‐P_4_ as catalyst, depolymerization of the polythioester P(**TCT**) (polymer sample according to Table 1, entry 2) afforded 21% **TCT** after 22 h (Table 2, entry 1). Other organic bases were subsequently evaluated for RCD (Tables 2 and S6) and the best results were achieved with TBD, enabling full conversion of the polymer after 24 h at 20°C and formation of **TCT** in 77% isolated yield (>99% conversion of P(**TCT**)). Monitoring of the depolymerization of P(**TCT**) with 0.5 equiv. DBU (Figure 5) showed that dispersity remained low. This suggests that RCD takes place through an unzipping pathway via backbiting from chain ends, rather than random chain scission, which would broaden dispersity. Interestingly, the peak maximum of the MW curve (*M*_p_) changes only marginally, from 16.1 kg mol^−1^ (initial polymer) to 15.7 kg mol^−1^ at 65% polymer conversion, indicating the unzipping length exceeds the initial polymer length of *n* = 98, that is, once RCD is initiated, polymer chains are fully converted to **TCT** and no termination is taking place (Table S7) [76, 77].

**TABLE 2:** RCD of P(**TCT**) in solution state with an organic base catalyst.

Entry	Cat.	Equiv.	Conv. of P(**TCT**)^b^ (%)	Sel. to **TCT** ^b^ (%)	Yield **TCT** ^b^ (%)
1^a^	^t^Bu‐P_4_	0.1	21	>99	21
2	TEA	0.1	<1	—	0
3	DBU	0.1	45	89	40
4	TBD	0.1	73	92	67
5	TBD	0.5	>99	—	77%^c^

Conditions: 0.2 M in DCM, 22–24 h, 20°C.

^a^ Solvent: toluene. Reaction in DCM resulted in 0% **TCT** formation, likely due to the slightly acidic methylene protons of DCM reacting with the superbase.

^b^ Conversion, selectivity and spectroscopic yield were based on the relative integrals in ^1^H‐NMR;.

^c^ Performed at 0.7 mmol polymer scale and the isolated monomer yield was determined after purification with flash column chromatography. Triethylamine (TEA), diazabicycloundecene (DBU), triazabicyclodecene (TBD).

**FIGURE 5:**
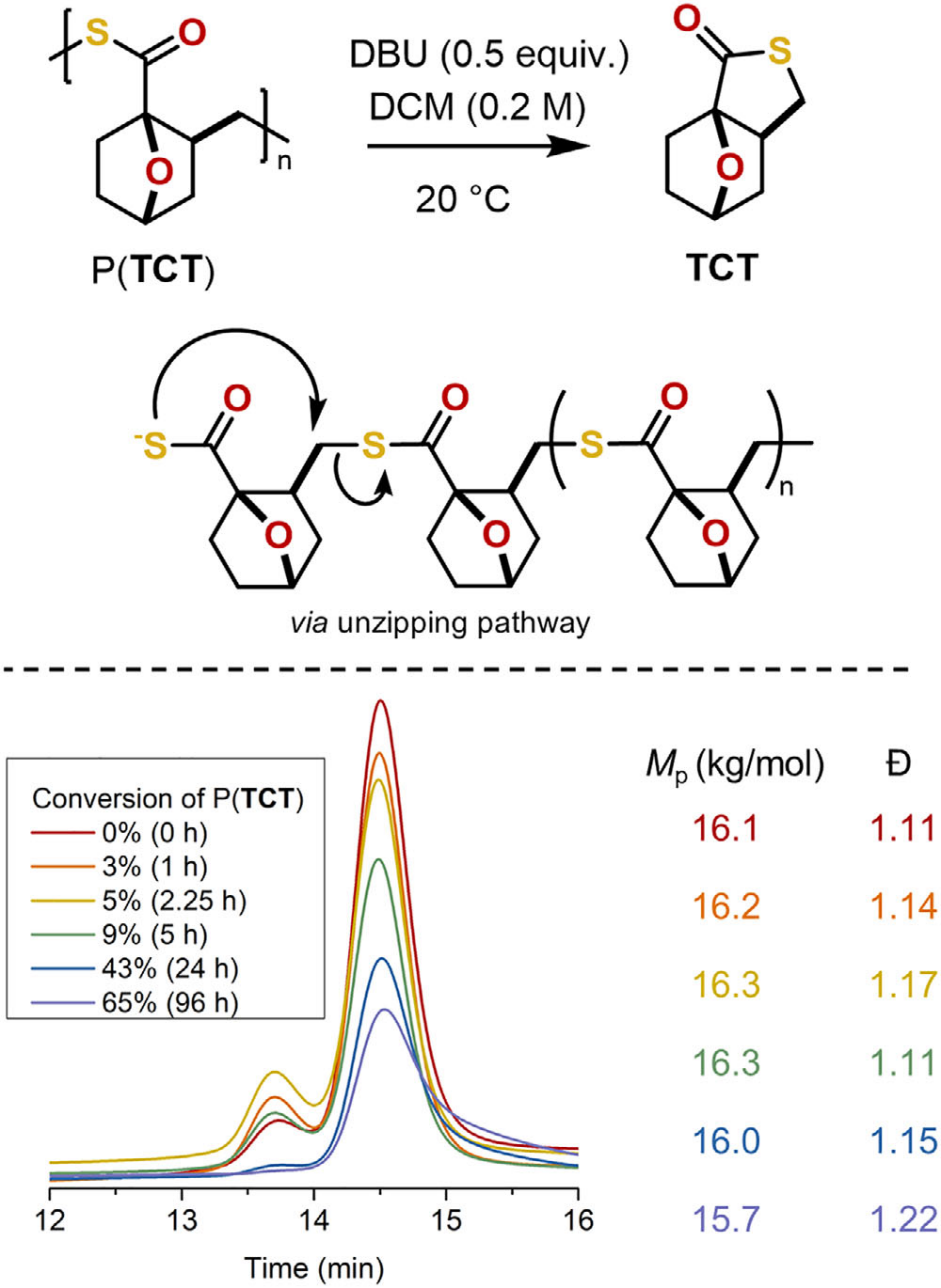
Base‐catalyzed RCD of P(**TCT**), normalized GPC traces and the backbiting/unzipping mechanism.

### 2.6 Depolymerization by Solvolysis

In addition to RCD, the polythioester could be depolymerized both via hydrolysis and methanolysis (Figure 6). When suspended under basic aqueous conditions, P(**TCT**) gradually reacted and was completely dissolved after three days, reaching 90% conversion after 160 h (Figure S7 and Table S8). As previously reported for P(TGBL) [27] hydrolysis proceeds via a 1,2‐addition‐elimination mechanism, that is, nucleophilic attack of hydroxide at the carbonyl carbon, forming the thiol‐carboxylic acid **TCT‐H_2_O**‐*2Na* as opposed to S_N_2‐type attack that would yield a hydroxy‐thiocarboxylic acid (Figure 6A). The formation of **TCT‐H_2_O**‐*2Na* as major product was confirmed by the low chemical shift of the methylene in the ^1^H (2.57, 2.00 ppm) and ^13^C NMR spectra (28.2 ppm), as well as the weak absorption at 2560 cm^−1^ from the S─H stretch vibration that was observed in IR after protonation and work‐up. Interestingly, upon prolonged hydrolysis, a side product slowly formed, reaching 14% after 7 days (Figure S7). It was identified as the disulfide product (**TCT**‐**H_2_O**)_ox_ formed from oxidative coupling of **TCT‐H_2_O**, as confirmed by a reaction of the isolated **TCT**‐**H_2_O** with iodine (Figure S48).

**FIGURE 6:**
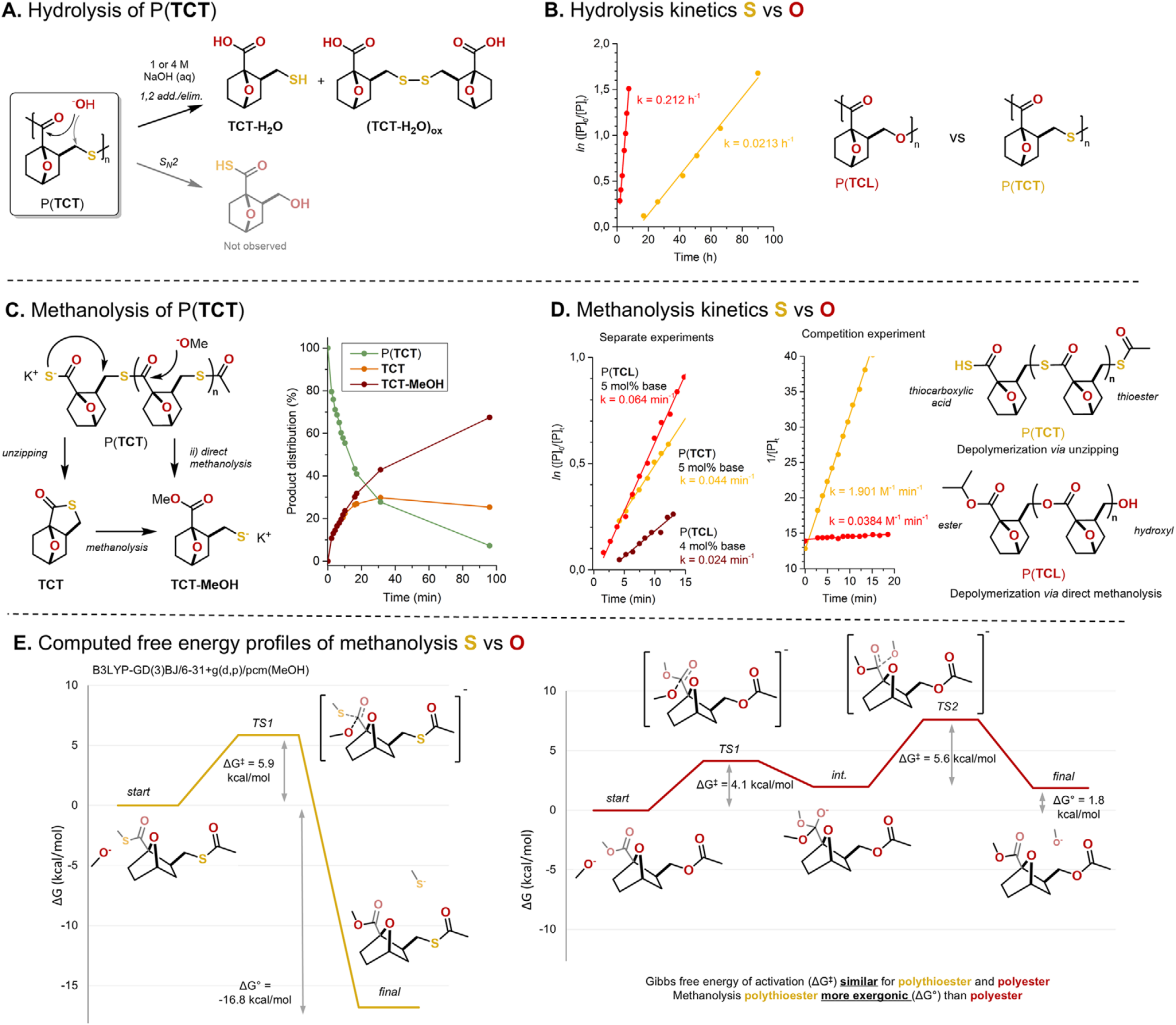
Depolymerization of P(**TCT**). (A) Reaction scheme and product distribution of hydrolysis of P(**TCT**) in basic aqueous conditions (suspended in NaOH 4 M in D_2_O); (B) Hydrolysis kinetics of P(**TCL**) and P(**TCT**), both suspended in NaOH 4 M in D_2_O (separate experiments); (C) Results of methanolysis of P(**TCT**) and product distribution indicating both i) backbiting to **TCT** followed by methanolysis and ii) direct methanolysis take place. (D) Comparison of polythioester and polyester methanolysis kinetics in separate experiments with CD_3_OD/CDCl_3_ v/v 1/1.5 0.1 M; base: K_2_CO_3_ reveals that methanolysis reaction rates are within the same order of magnitude, whereas the competition experiment with CD_3_OD/CDCl_3_ v/v 1/2.22; 0.078 M each; 5 mol% K_2_CO_3_ indicates polythioester undergoes depolymerization mainly through unzipping; **E**) The computed free energy profiles of methanolysis for P(**TCT**) and P(**TCL**) at 25°C show ΔΔG^‡^ = < 1 kcal mol^−1^.

Next, the hydrolysis kinetics of the polythioester and its polyester analogue, P(**TCL**), were compared (Table S9). The semilogarithmic plots (Figure 6B) revealed first‐order kinetics in polymer concentration for both systems. Unexpectedly, hydrolysis of P(**TCT**) was found to be 10× slower than P(**TCL**). Typically, solvolysis of polythioesters is faster under alkaline conditions [28], however, as both polymers were suspended under basic aqueous conditions, these apparent rate differences may arise from variations in solubility, hydrophilicity, or particle size, rather than from the differences in intrinsic reactivity.

To enable a direct comparison of intrinsic solvolysis rates under homogeneous conditions, methanolysis was investigated in organic solvent. The reaction with 5 mol% K_2_CO_3_ as base catalyst in a deuterated methanol/chloroform mixture was monitored by ^1^H‐NMR (Figure 6C,D and Table S11). The immediate production of **TCT‐MeOH** (from direct methanolysis) as well as **TCT** (the backbiting product) indicated that under these conditions both routes to the methanolized product take place. The reaction followed pseudo first‐order kinetics with respect to P(**TCT**) concentration (k_obs_ = 0.044 min^−1^). Under the same conditions, methanolysis of the polyester P(**TCL**) proceeded somewhat faster (k_obs_ = 0.064 min^−1^, Figure 6D and Table S12). It was hypothesized that the thiocarboxylic acid end‐group (pK_a_ ≈ 3) of P(**TCT**) (n ≈ 130) partially neutralizes the base catalyst (ca. 0.8 mol%, pK_a_ ≈ 10), effectively reducing the catalyst loading to 4.2 mol%. In contrast, the polyester, being capped with an ester and hydroxyl, lacks acidic end‐groups and consequently, differences in effective catalyst concentration complicated direct comparison (see methanolysis of polyester with 4 mol% base, Figure 6D and Table S13). Additionally, the thiol functionality (pK_a_ ≈ 10) of the formed **TCT‐MeOH** may further quench the base after the initial reaction stage. A direct competition experiment between P(**TCT**) and P(**TCL**) showed that polyester depolymerization proceeded only to 4% conversion before ceasing, whereas the P(**TCT**) continued to depolymerize (Figure 6D and Table S14). Presumably, once **TCT‐MeOH** formed, the remaining base was quenched thereby halting further polyester methanolysis. In contrast, the polythioester continued to depolymerize via intramolecular backbiting, followed by methanolysis.

These findings support that P(**TCT**) depolymerizes predominantly through backbiting of the thiocarboxylate chain end, which is regenerated after each ring‐closing event and therefore sustains unzipping without consuming base. The distinguishing factor is the acidity of the chain end: only the thiocarboxylic acid (pK_a_ ≈ 3) is deprotonated under these conditions to a nucleophile capable of intramolecular attack, while the hydroxyl end‐group of P(**TCL**) is less nucleophilic and does not backbite under these conditions.

DFT calculations indeed indicate a negligible difference in the Gibbs free energy of activation (ΔΔG^‡^ = < 1 kcal mol^−1^) between the direct basic methanolysis pathways of the ester and thioester (Figure 6E; Table S16 and S17). It should be noted that these calculations were performed using methoxide as the nucleophile, whereas under the experimental conditions (K_2_CO_3_ in methanol) methanolysis is expected to proceed via base‐assisted activation of methanol rather than via the free methoxide. Nevertheless, the small difference in activation energy for this simplified mechanism supports that the enhanced methanolysis observed for the polythioester likely does not arise from intrinsic differences in activation barrier for direct solvolysis, but rather from the additional backbiting pathway as experimentally observed.

Consistent with expectations, thiolate expulsion was calculated to be much more exergonic compared to methoxide expulsion, and the tetrahedral intermediate formed during thioester methanolysis could not be optimized to a stable minimum but instead collapses directly to the ester and thiolate products. This behavior reflects the superior leaving‐group ability of the thiolate anion relative to alkoxide, which renders the tetrahedral intermediate intrinsically unstable and prevents its localization as a minimum on the computed potential energy surface.

The comparable methanolysis rates indicate that the intrinsic susceptibility of the ester and thioester linkages toward nucleophilic attack is similar, suggesting that the substantially slower hydrolysis of P(**TCT**) in aqueous media is largely governed by physical factors associated with the heterogeneous reaction conditions.

## 3 Conclusion

In summary, we have developed a tricyclic oxanorbornane‐fused thionolactone (**TCTn**) that undergoes rapid and highly selective IROP using either phosphazene (^t^Bu‐P_4_) catalyst or tetrabutylammonium thioacetate (NBu_4_SAc) initiator to yield high MW polythioester P(**TCT**) (ca. 100 kg mol^−1^). The resulting polythioester exhibits a *T*_g_ of 171°C, placing it among the highest reported for polythioesters from thio(no)lactone (I)ROP. Notably, P(**TCT**) surpassed the *T*_g_ of its polyester analogue, P(**TCL**), deviating from the commonly observed trend that O→S substitution lowers *T*_g_ through increased backbone flexibility. These findings underscore the importance of rigid, multicyclic monomer design for accessing high *T*_g_ sustainable materials.

We show that prolonged polymerization times result in RCD via backbiting to the thiolactone **TCT**. Also, under mild basic conditions, the isolated polythioester efficiently unzips back to **TCT**. The low‐strain thiolactone **TCT** does not undergo ROP and DFT calculations confirm that ROP of **TCT** to the polythioester is indeed endergonic. This behavior prevents closed‐loop recycling for this system, and emphasizes the importance of the IROP pathway to overcome the low driving force of thiolactones relative to lactones.

Furthermore, we present one of the few direct comparisons of thermal and solvolysis behavior of polyester versus polythioester. With respect to the latter, suspended in aqueous basic conditions P(**TCT**) hydrolyzes slower than its polyester analogue. In contrast, under homogeneous conditions, methanolysis proceeds through both direct solvolysis and backbiting with a rate comparable to the polyester depolymerization, which occurs only via direct methanolysis. Therefore, susceptibility of the ester and thioester toward nucleophilic attack is similar. A competition experiment further suggested any enhanced solvolysis of the polythioester arises from the backbiting mechanism rather than intrinsic differences in activation energy, consistent with DFT computed transition states.

Overall, this study provides insights into the design of sustainable, sulfur‐containing polymers. It highlights the intricacies that come with O→S substitution, including byproduct formation arising from sulfur chemistry such as S─S coupled polymer chains, and monomeric species, and revealing a *T*_g_ unexpectedly higher than that of the polyester. Furthermore, it demonstrates that the apparent difference in degradation behavior between this polyester and polythioester is governed by end‐group chemistry and backbiting.

## Author Contributions

**Eva Harsevoort**: conceptualization, investigation, writing – original draft, methodology, visualization, formal analysis, data curation. **Henk‐Jan Akkerman**: investigation, methodology, writing – review and editing. **Martin Lutz**: investigation, writing – review and editing, formal analysis. **Arnaud Thevenon**: conceptualization, writing ‐ review and editing, visualization, methodology, supervision, resources, funding acquisition. **Pieter C. A. Bruijnincx**: conceptualization, funding acquisition, writing – review and editing, methodology, project administration, supervision, resources.

## Conflicts of Interest

The authors declare no conflicts of interest.

## Supporting information




**Supporting File**: Experimental details, characterizations and additional optimization tables are available free of charge in the Supporting Information. Additional spectroscopic, thermoanalytic, chromatographic, and computational data files that support the findings of this study are openly available in the Yoda data repository at https://doi.org/10.24416/UU01‐HFNF28. CCDC 2543058 contains the supplementary crystallographic data for this paper. The authors have cited additional references within the Supporting Information [78, 79, 80, 81, 82, 83, 84, 85, 86].

## Data Availability

The data that support the findings of this study are openly available in Yoda at https://doi.org/10.24416/UU01‐HFNF28.
