# Supplementary material for: High‐T g Polythioesters From Isomerization Ring‐Opening Polymerization of a Renewable Oxanorbornane‐Fused Thionolactone
Source: Angew Chem Int Ed Engl. 2026 Sep 4;65(43):e6168208. doi: 10.1002/anie.6168208 (PMC13676387; doi:10.1002/anie.6168208)
Supplement: Supplementary file 1 [file ANIE-65-e6168208-s001.docx]

Electronic Supporting Information for

High-*T*_g_ Polythioesters from Isomerization Ring-Opening Polymerization of a Renewable Oxanorbornane-Fused Thionolactone

Eva Harsevoort,^a^ Henk-Jan Akkerman,^a^ Martin Lutz,^b^ Arnaud Thevenon,^a,*^ Pieter C. A. Bruijnincx^a,*^

a. Organic Chemistry and Catalysis, Institute for Sustainable and Circular Chemistry, Faculty of Science, Utrecht University, Universiteitsweg 99, 3584 CG Utrecht, The Netherlands.

b. Structural Biochemistry, Bijvoet Centre for Biomolecular Research, Faculty of Science, Utrecht University, Universiteitsweg 99, 3584 CG Utrecht, The Netherlands.

* Corresponding Authors. Email: a.a.thevenon-kozub@uu.nl; p.c.a.bruijnincx@uu.nl.

Contents

[General considerations	2](#_Toc237355698)

[Materials, reagents, and methods	2](#_Toc237355699)

[Spectroscopic characterizations	2](#_Toc237355700)

[Molecular weight measurements	3](#_Toc237355701)

[Thermal analysis	3](#_Toc237355702)

[Procedures, Results and Characterization	4](#_Toc237355703)

[Monomer synthesis	4](#_Toc237355704)

[X-ray crystal structure determination of TCTn	5](#_Toc237355705)

[Polymerization procedures	5](#_Toc237355706)

[Polymerization results	8](#_Toc237355707)

[Polymer characterization	10](#_Toc237355708)

[Ring-closing depolymerization	12](#_Toc237355709)

[Hydrolysis	13](#_Toc237355710)

[Methanolysis	17](#_Toc237355711)

[Computational details	24](#_Toc237355712)

[Ring opening	24](#_Toc237355713)

[Methanolysis	25](#_Toc237355714)

[Spectra	27](#_Toc237355715)

[Monomeric species	27](#_Toc237355716)

[Polymer spectra	42](#_Toc237355717)

[Spectra - polymerization profile	71](#_Toc237355718)

[IROP catalyzed by ^t^Bu-P_4_	71](#_Toc237355719)

[Polymerization catalyzed by NBu_4_SAc at 80 °C	76](#_Toc237355720)

[Polymerization catalyzed by NBu_4_SAc at 50 °C	80](#_Toc237355721)

[Spectra – depolymerization	83](#_Toc237355722)

[References	87](#_Toc237355723)

**Associated content:** CCDC 2543058 contains the supplementary crystallographic data for this paper. Additional spectroscopic, thermoanalytic, chromatographic, and computational data files that support the findings of this study are openly available in the Yoda data repository at https://doi.org/10.24416/UU01-HFNF28.

# General considerations

## Materials, reagents, and methods

Unless stated otherwise, all solvents and commercially available reagents were used as purchased. Molecular sieves (4 Å) were activated thermally prior to use (250 °C, several days). Reactions requiring inert conditions were carried out under a nitrogen (N_2_) atmosphere and with dried glassware, using Schlenk techniques or in an M. Braun glovebox. Toluene (purchased from VWR Chemicals) and dichloromethane (DCM, purchased from VWR Chemicals) were collected from an M. Braun MB-SPS 800 solvent purification system, degassed by purging with N_2_, and further dried on 4 Å molecular sieves. Methanol was purchased from VWR Chemicals. *N,N*-Dimethylacetamide (DMA, purchased from Acros) was distilled over CaH_2_, purged with N_2_ and stored on 4 Å molecular sieves. Potassium thioacetate (KSAc), triethylamine (TEA) was purchased from Acros. Lawesson’s reagent (LR) and 1,8-diazabicyclo[5.4.0]undec-7-ene (DBU) were purchased from TCI. Anhydrous benzyl alcohol (BnOH, purchased from Merck) was degassed by purging with N_2_ and dried on 4 Å molecular sieves. Phosphazene base 1-*tert*-butyl-4,4,4-tris(dimethylamino)-2,2-bis[tris(dimethylamino)phosphoranylidenamino]-2λ^5^,4λ^5^-catenadi(phosphazene)] (^t^Bu-P_4_) as 0.8 M solution in hexane and 1,5,7-triazabicyclo[4.4.0]dec-5-ene (TBD) were purchased from Merck. Lactone **TCL** was synthesized according to previously reported work.[1] Tetrabutyl-ammonium thioacetate (NBu_4_SAc) was synthesized according to literature[2] and stored in the glovebox freezer (-40 °C).

## Spectroscopic characterizations

**Nuclear magnetic resonance** (NMR) spectra were recorded on a Varian VNMRS-400 or a Jeol JNM-ECZL G 400MHz spectrometer at 25 °C. Chemicals shifts (δ) are reported relative to TMS with the residual solvent signal,[3] expressed in ppm, and coupling constants (J) are quoted in hertz (Hz). Peak multiplicity is described as s (singlet), d (doublet), t (triplet), q (quartet), br (broad singlet) and m (multiplet) or combinations thereof.

**Attenuated Total Reflection Fourier Transform Infrared** (ATR-FTIR) spectra were recorded on a PerkinElmer Frontier FTIR spectrometer, equipped with the universal ATR sampling station. Prior to each measurement a background spectrum consisting of 8 scans was collected and subsequently subtracted from the sample spectrum. Intensities are reported with w (weak), m (medium), s (strong), b (broad) and combinations thereof. The sample was placed onto the sample plate, ensuring total coverage of the ATR crystal, and was pressed by the toner arm.

**Electrospray Ionization** (ESI) mass spectrometry was carried out using an Agilent technologies 6560 ion mobility Q-TOF instrument.

**UV-Vis** absorption spectra were obtained on 25 mM solution in DCM in quartz cuvettes using a LAMBDA 365 UV/Vis Spectrophotometer.

## Molecular weight measurements

**Gel Permeation Chromatography** (GPC) measurements were carried out on a Waters Alliance 2695 system with a Waters 2789 UV detector, a 2414 RI detector, two Agilent PL MIXED-D columns in series, equipped with a precolumn. Chloroform (Biosolve) was used as the eluent, the measurements were run at a flow rate of 1 mL/min at 30 °C and analyzed with Empower 3 software. For calibration polystyrene standards (PSS) for Mixed-D columns were used and measured for each sample batch. The samples were dissolved (3 mg polymer/mL) in the eluent and filtered through a 0.45 µm syringe filter into the GPC vials.

## Thermal analysis

**Thermogravimetric analysis** (TGA) measurements were performed on a TA instruments TGA Q50 Thermographic Analyzer under nitrogen flow on a platinum pan and analyzed using TA instruments *Universal Analysis 2000*. The thermal degradation was investigated using samples of ca 4 mg loaded onto the pan at a heating rate of 10 °C/min from rt to 600 °C. From the TGA and DTG (derivative thermogravimetric) traces the temperature at 5% and 50% weight loss (*T*_5%_ and *T*_50%_), temperature at maximum weight loss (*T*_max_), and onset temperature (*T*_onset_) were determined, where the *T*_onset_ was determined from the intersection of baseline and the tangent at maximum gradient.

**Modulated Differential Scanning Calorimetry** (MDSC) measurements were performed on a TA instruments Discovery DSC and analyzed with the TA instruments *TRIOS* software (version 5.7.1). Tzero low mass aluminium pans fitted with a Tzero aluminium lid were loaded with approximately 3 mg of sample. The modulation rate was set to 1 °C/min. The first heating scan had a heating rate of 10 °C/min to 200 °C, the sample was held isothermally for 2 min before cooling down at 3 °C/min to 20 °C, where it was held isothermally for 5 min. The glass transition temperatures (*T*_g_) were recorded on the second heating scan, which had a heating rate of 3 °C/min to 200 °C.

For the solid RCD experiments in the DSC, a pan was loaded with ca. 2 mg of polymer, heated to 155 °C at a rate of 10 °C/min. Upon reaching 155 °C the polymer was held at an isothermal at the times displayed in Table S5.

# Procedures, Results and Characterization

## Monomer synthesis

**Thionolactone TCTn:**In a N_2_ glovebox, a 100 mL pressure tube was charged with lactone **TCL** (500 mg, 3.24 mmol, 1 equiv.), Lawesson’s reagent (LR) (1.06 g, 2.61 mmol, 0.8 equiv.) and toluene (20 mL). The resulting yellow suspension was heated to 150 °C outside the glovebox. After 21 h the dark brown solution was cooled to rt and an aliquot of 0.2 mL was dried *in vacuo* and dissolved in CDCl_3_ for ^1^H-NMR analysis, which indicated 80% conversion of **TCL**, with full selectivity towards the product. The crude reaction mixture was then filtered over an acidic alumina plug, which was flushed with a mixture of petroleum ether/ethyl acetate (50/50 v/v, 2x 80 mL). The filtrate was then concentrated *in vacuo* and the resulting brown oil was purified by silica gel flash column chromatography (5 to 10 v/v% ethyl acetate in petroleum ether), affording the product as a light-yellow oil (372 mg, 2.18 mmol, 67% yield). Prior to polymerization, **TCTn** was crystallized from slow evaporation of diethyl ether and heptane, yielding yellow crystals.

**TLC** (ethyl acetate/petroleum ether, 50/50, v/v) R_f_ = 0.60; **^1^H-NMR** (400 MHz, CDCl_3_) δ 4.88 (p, *J* = 2.5 Hz, 1H, H4), 4.71 (t, *J* = 8.9 Hz, 1H, H1a), 4.17 (t, *J* = 9.3 Hz, 1H, H1b), 2.80 (tt, *J* = 9.0, 5.8 Hz, 1H, H6), 2.22 (td, *J* = 12.1, 4.9 Hz, 1H, H2a), 2.11 – 1.99 (m, 1H, H3a), 1.98 – 1.91 (m, 2H, H5), 1.81 (ddd, *J* = 12.0, 9.0, 3.6 Hz, 1H, H2b), 1.64 (ddd, *J* = 11.9, 9.0, 5.0 Hz, 1H, H3b) ppm; **^13^C{^1^H}-NMR** (100 MHz, CDCl_3_) δ 215.8 (C8), 95.6 (C1), 81.3 (C4), 79.8 (C7), 46.4 (C6), 37.7 (C5), 31.5 (C2), 29.9 (C3) ppm; **IR (ATR)**: ṽ = 2984 (m), 2954 (m), 2874 (w), 1784 (w), 1693 (w), 1467 (w), 1367 (m), 1329 (m), 1298 (m), 1249 (s), 1209 (s), 1189 (s), 1168 (s), 1084 (m), 1006 (m), 949 (m), 912 (m), 887 (m), 820 (m), 797 (m), 633 (w), 555 (m) cm^-1^; **ESI-MS:** *m/z* [*M*+MeOH+Na]^+^ calculated for C_9_H_14_O_3_SNa: 225.0561, found: 225.0545.

**Thiolactone TCT**:Lactone **TCL** (530 mg, 3.44 mmol, 1 equiv.) and potassium thioacetate (791 mg, 6.92 mmol, 2 equiv.) were dissolved in *N,N*-dimethylacetamide (43 mL, [**TCL**]_0_ = 0.08 M) and the dark-brown solution was stirred at 160 °C for 24 h after which ^1^H-NMR analysis showed full conversion of **TCL**. After cooling the crude reaction mixture to rt, water (175 mL) was added, and the product was extracted from the aqueous layer with ethyl acetate. The organic layers were dried using Na_2_SO_4_ and filtered. ^1^H-NMR analysis of the dried filtrate indicated mostly *N,N*-dimethylacetamide and potassium thioacetate were present so the dried filtrate was partitioned between ethyl acetate (50 mL) and water (50 mL). The organic layer was washed with saturated NaCl solution (2 mL), dried with Na_2_SO_4_, filtered and evaporated *in vacuo*. The dried crude was dissolved in DCM, precipitating potassium thioacetate, and purified using silica gel flash column chromatography (0 to 25 v/v% ethyl acetate in petroleum ether), affording the product (32.4 mg, 0.19 mmol, 5% yield).

The same compound was also isolated from depolymerization of P(**TCT**) by ring-closing depolymerization (see ring-closing depolymerization section below).

**TLC** (ethyl acetate/petroleum ether, 50/50 v/v) R_f_ = 0.75; **^1^H-NMR** (400 MHz, CDCl_3_) δ 4.88 (t, *J* = 4.9 Hz, 1H, H4), 3.30 (dd, *J* = 10.9, 8.1 Hz, 1H, H7a), 3.13 (dd, *J* = 10.9, 9.5 Hz, 1H, H7b), 2.82 – 2.70 (m, 1H, H6), 2.12 – 1.99 (m, 1H, H2a), 1.99 – 1.81 (m, 3H, H3a and H5), 1.69 – 1.56 (m, 2H, H2b and H3b) ppm; **^13^C{^1^H}-NMR** (100 MHz, CDCl_3_) δ 202.7 (C8), 93.1 (C1), 81.1 (C4), 48.7 (C6), 40.0 (C5), 36.3 (C7), 30.0 (C3), 28.6 (C2) ppm; **IR (ATR)**: ṽ = 3366 (w), 2959 (m), 2923 (m), 2853 (m), 1734 (m), 1688 (m), 1452 (m) 1435 (m), 1297 (m), 1260 (m), 1187 (m), 1029 (m), 1011 (m), 931 (m), 867 (m), 827 (m), 788 (m), 645 (m) cm^-1^; **ESI-MS:** *m/z* [*M*+H]^+^ calculated for C_8_H_11_O_2_S: 171.0480, found: 171.0488.

## X-ray crystal structure determination of TCTn

Crystals of thionolactone **TCTn** were grown by slow evaporation of a solution in diethyl ether and heptane. C_8_H_10_O_2_S, Fw = 170.22, pale yellow needle, 0.44 × 0.19 × 0.07 mm^3^, monoclinic, P2_1_/c (no. 14), a = 20.5764(7), b = 5.7256(2), c = 14.1334(5) Å, β = 106.635(2) °, V = 1595.40(10) Å^3^, Z = 8, D_x_ = 1.417 g/cm^3^, μ = 0.35 mm^-1^. The diffraction experiment was performed on a Bruker Kappa ApexII diffractometer with sealed tube and Triumph monochromator (λ = 0.71073 Å) at a temperature of 150(2) K up to a resolution of (sin θ/ λ)_max_ = 0.65 Å^-1^. The crystal appeared to be twinned with a twofold rotation about *uvw*=[0,0,1] as twin operation. Because of this twinning, two orientation matrices were used for the intensity integration with the Eval15 software[4]. A multi-scan absorption correction and scaling was performed with TWINABS[5] (correction range 0.70-0.75). A total of 48766 reflections was measured, 3686 reflections were unique (R_int_ = 0.032), 3329 reflections were observed [I>2σ(I)]. The structure was solved with dual-space methods using SHELXT.[6] Structure refinement was performed with SHELXL-2019[7] on F^2^ of all reflections. Non-hydrogen atoms were refined freely with anisotropic displacement parameters. All hydrogen atoms were located in difference Fourier maps and refined with a riding model. 200 Parameters were refined with no restraints. R1/wR2 [I > 2σ(I)]: 0.0341 / 0.0942. R1/wR2 [all refl.]: 0.0382 / 0.0962. S = 1.075. Twin fraction BASF=0.0500(5). Residual electron density between ‑0.23 and 0.39 e/Å^3^. Geometry calculations and checking for higher symmetry was performed with the PLATON program.[8]

CCDC 2543058 contains the supplementary crystallographic data for this paper. These data can be obtained free of charge from The Cambridge Crystallographic Data Centre via www.ccdc.cam.ac.uk/data_request/cif.

## Polymerization procedures

Monomer conversion was determined from the ratio of integrals in NMR from the resonances of polymer (P(**TCT**)), monomer (**TCTn**), and (when applicable) the thiolactone side product (**TCT**). A worked-out example of conversion and selectivity determination is given below (Figure S4). Crude NMR spectra used for conversion and selectivity determination can be found on the data repository (as well as all raw data files).

Full characterization with NMR (^1^H-^1^H COSY, ^1^H-^13^C ASAP-HMQC, ^1^H-^13^C HMBC) of the polythioester was done on a low molecular weight polymer sample, synthesized with [**TCTn**]/[^t^Bu-P_4_]/[BnOH] = 20/1/1, according to Table S1, Entry S1. See Figure S52-S56 for assigned spectra of P(**TCT**) synthesized with ^t^Bu-P_4_ and Figure S78-79 for assigned spectra of P(**TCT**) synthesized with NBu_4_SAc.

Polymerizations were carried out on 0.3 to 3 mmol scale. Procedure examples are for **TCTn**/Cat. 100/1 at 1.0 mmol scale. For other targeted molecular weights, the amount of catalyst was scaled, while keeping [**TCTn**]_0_ consistent at 1.0 M.

**Using ^t^Bu-P_4_ as catalyst**

In a N_2_ glovebox a microwave vial or Schlenk flask was charged with **TCTn** (170.2 mg, 1.00 mmol, 1.00 equiv.) and dry toluene (0.9 mL). The required amount of the ^t^Bu-P_4_ stock solution (0.8 M in hexane) as indicated in Table S1, was dried *in vacuo*. A BnOH initiator stock solution (0.1 M in toluene) was prepared, and the required amount (for ^t^Bu-P_4_/BnOH 1/1) was added to the dried catalyst. Outside of the glovebox, the **TCTn** solution (light yellow) was stirred at 80 °C for 10 min, after which the ^t^Bu-P_4_/BnOH mixture was added (0.1 mL of 0.1 M solution, 0.01 mmol, 0.01 equiv.), with [**TCTn**]_0_ = 1.0 M. After the indicated time, the reaction was quenched by adding 1-2 mL of technical grade chloroform. An aliquot was taken for ^1^H-NMR analysis in CDCl_3_ or CD_2_Cl_2_ to determine the **TCTn** conversion and ratio P(**TCT**):**TCT**. The quenched solution was concentrated, redissolved in a minimal amount of DCM, and precipitated from methanol (4 mg polymer per mL methanol). The white suspension was then centrifuged at 10000 RPM for 10 min and decanted. The polymer was washed with methanol (1 mL per 4 mg polymer) and centrifuged and decanted once again, and dried *in vacuo*, producing a white powder.

Catalyst/polymer separation issues were encountered with polymers formed with ^t^Bu-P_4_. Repeated precipitation was required to remove residual catalyst after which, though clean according to NMR analysis, still low decomposition temperatures were observed. For P(**TCT**) obtained using ^t^Bu-P_4_ the decomposition temperature was reduced considerably by approximately 90 °C (*T*_5%_ = 167.6 °C, Figure S67), a drop attributed to residual catalyst. Repeated precipitation still resulted in a two-stage degradation (*T*_max_ = 213 and 331 °C, Figure S68). We hypothesize that residual catalyst present in the polymer induces degradation through RCD, with the first mass loss corresponding to the evaporation of thiolactone **TCT**, (*T*_max)_ = 175 °C for the isolated thiolactone, Figure S35). Indeed, when heating a solid polymer sample containing 0.6 mol% residual ^t^Bu-P_4_ catalyst in the DSC to 155 °C for different times, RCD was observed as confirmed by ^1^H-NMR, and complete conversion of P(**TCT**) was observed after 90 min at 155 °C (Table S5).

**Using NBu_4_SAc as catalyst**

In a N_2_ glovebox a microwave vial was charged with **TCTn** (170.2 mg, 1.00 mmol, 1.00 equiv.) and *N,N*-dimethylacetamide (0.33 mL) was added to make a 3.0 M solution. A 0.0145 M NBu_4_SAc stock solution was prepared. Outside of the glovebox, the microwave vial was heated to the desired temperature for 10 min, after which 0.67 mL (containing 0.01 mmol initiator, 0.01 equiv.) of the NBu_4_SAc stock solution was added to the monomer solution ([**TCTn**]_0_ = 1 M). After the indicated time, the reaction was quenched with acetic acid, and an aliquot was taken for ^1^H-NMR analysis in CDCl_3_ or CD_2_Cl_2_ to determine the **TCTn** conversion and ratio P(**TCT**)/**TCT**. The quenched solution was concentrated, redissolved in a minimal amount of DCM, and precipitated from methanol (4 mg polymer per mL methanol). The white suspension was then centrifuged at 10000 RPM for 10 min and decanted. The polymer was washed with methanol (1 mL per 4 mg polymer) and centrifuged and decanted once again, and dried *in vacuo*, producing a white powder.

**Reducing S-S bonds in polymer**

A polymer sample formed according to Table S1, Entry 4 (11 mg, 0.064 mmol repeat unit, 0.00016 mmol end group) was dissolved in 2 mL CHCl_3_ and 12 mg polystyrene with M_p_ 2340 g/mol was added as internal standard. The solution was split into two equal parts and to one part reducing reagent dithiothreitol (DTT) was added (10 mg, 0.064 mmol, 1 equiv w.r.t. repeat unit, 405 equiv w.r.t end group) was added. Both samples were measured with GPC.

Figure S1: Mechanisms for thionolactone ROP and IROP.

## Polymerization results

Table S1: Isomerization ring-opening polymerization (IROP) of thionolactone **TCTn**.^[a]^

| Entry | Cat. | **TCTn** loading vs Cat. | Time | Sel. to P(**TCT**)^[b]^ (%) | Isolated yield P(**TCT**) (%) | M_n,calc_  (kg mol^-1^)^[c]^ | M_n,NMR_  (kg mol^-1^)^[d]^ | M_n,GPC_  (kg mol^-1^)^[e]^ | Đ^[f]^ (% high MW chains) | T_d,max_ (°C) | T_d,5%_ (°C) | T_g_ (°C) |
| --- | --- | --- | --- | --- | --- | --- | --- | --- | --- | --- | --- | --- |
| S1 | ^t^Bu-P_4_/  BnOH | 20 | 10 min | 91 | - | - | 7.5 | 9.1 | 1.26 | - | - | - |
| 1 | ^t^Bu-P_4_/  BnOH | 100 | 10 min | 98 | 78 | 16.8 | - | 25.0 | 1.22 (37.7) | 207.0 | 184.1 | 166.4 |
| S2 | ^t^Bu-P_4_/  BnOH | 100 | 2 h | - | - | - | - | 25.1 | 1.17 (20.0) | 234.3 (213.4  331.5)^[h]^ | 167.6 (209.2) | (164.1) |
| 2 | NBu_4_SAc | 100 | 1 h | 96 | 54 | 16.4 | 22.2 | 21.7 | 1.08 (8.0) | 294.7 | 255.7 | 167.3 |
| 3 | NBu_4_SAc | 200 | 1 h | 94 | 79 | 32.1 | 36.0 | 37.6 | 1.17 (23.9) | 313.7 | 272.7 | 169.4 |
| 4 | NBu_4_SAc | 400 | 2 h^[g]^ | 94 | 40 | 64.1 | 69.7 | 64.2 | 1.31 (38.8) | 303.9 | 277.9 | 171.3 |
| 5 | NBu_4_SAc | 600 | 3 h | 95 | 87 | 97.1 | 103.2 | 75.3 | 1.34 (36.6) | 309.0 | 261.2 | 171.7 |
| 6 | NBu_4_SAc | 800 | 3 h | 94 | 73 | 128.1 | 131.5 | 91.9 | 1.39 (48.0) | 305.1 | 272.0 | 171.3 |

[a] For IROP conditions, see general procedures. Polymerizations were conducted at 80 °C. Conversion of **TCTn** was >99% for all entries. [b] Selectivity to polymer, with **TCT** as sole side product. [c] M_n,calc_ calculated as conversion $\times$ selectivity $\times$ [**TCTn**]/[Init.] $\times$ MW of monomer + MW of initiator; [d] M_n,NMR_ as determined from chain length n based on end-group analysis × MW monomer + MW initiator; [e] M_n,GPC_ determined by gel permeation chromatography in chloroform, calibrated with polystyrene standards; [f] Đ = M_w,GPC_/M_n,GPC_. All samples exhibit bimodal MW distributions; the percentage of high MW signal from the GPC integral is depicted in brackets; [g] 98% conversion of **TCTn** was measured after 1 h. [h] Repeated precipitated improved T_d_, see Figures S67-S68.

Table S2: Results of IROP catalyzed by ^t^Bu-P_4_ with various reaction times.^[a]^

| Entry | Time | Residual **TCTn** (%)^[b]^ | P(**TCT**) (%)^[b]^ | **TCT** (%)^[b]^ | Sel. to P(**TCT**) (%)^[c]^ | M_n,GPC_  (kg mol^-1^) ^[d]^ | Đ^[e]^ | GPC area of high MW peak (%) | [M] (mol/L) |
| --- | --- | --- | --- | --- | --- | --- | --- | --- | --- |
| 1 | 0.5 min | 24.0 | 75.2 | 0.8 | 98.9 | 21.1 | 1.07 | - | 0.240 |
| 2 | 1 min | 10.5 | 88.5 | 1.0 | 98.9 | 27.9 | 1.11 | 11 | 0.105 |
| 3 | 10 min | 0 | 97.7 | 2.2 | 97.7 | 25.1 | 1.19 | 32 | 0 |
| 4 | 2 h | 0 | 93.5 | 6.5 | 93.5 | 33.9 | 1.24 | 14 | 0 |
| 5 | 21 h | 0 | 74.6 | 25.4 | 74.6 | - | - | 12 | 0 |
| 6 | 46 h | 0 | 8.6 | 91.4 | 8.6 | - | - | - | 0 |

[a] For IROP conditions, see general procedure. Polymerizations were conducted at 80 °C. [b] Determined from the relative integrals in ^1^H-NMR. [c] Selectivity to polymer, with **TCT** as sole side product. [d] M_n,GPC_ determined by gel permeation chromatography in chloroform, calibrated with polystyrene standards; [e] Đ = M_w,GPC_/M_n,GPC_.

Figure S2: Ring-closing depolymerization observed after prolonged reaction time.

Table S3: Product distribution results of IROP catalyzed by NBu_4_SAc at 80 °C with various reaction times.^[a]^

| Entry | Time (min) | Residual **TCTn**(%)^[b]^ | P(**TCT**) (%)^[b]^ | **TCT** (%)^[b]^ | Sel. to P(**TCT**) (%)^[c]^ | M_n,GPC_  (kg mol^-1^) ^[d]^ | Đ^[e]^ | GPC area of high MW peak (%) | [M] (mol/L) | ln([M]_0_/[M]_t_) |
| --- | --- | --- | --- | --- | --- | --- | --- | --- | --- | --- |
| 1 | 1 | 65 | 33 | 2 | 94 | 5.9 | 1.10 | 5 | 0.65 | 0.431 |
| 2 | 5 | 14 | 81 | 5 | 94 | 11.6 | 1.09 | 6 | 0.14 | 1.966 |
| 3 | 10 | 3 | 91 | 6 | 94 | 16.6 | 1.11 | 9 | 0.03 | 3.507 |
| 4 | 30 | 0 | 96 | 6 | 96 | 16.0 | 1.14 | 8 | 0 | - |

[a] For IROP conditions, see general procedure. Polymerizations were conducted at 80 °C. [b] Determined from the relative integrals in ^1^H-NMR. [c] Selectivity to polymer, with **TCT** as sole side product. [d] M_n,GPC_ determined by gel permeation chromatography in chloroform, calibrated with polystyrene standards; [e] Đ = M_w,GPC_/M_n,GPC_.

Table S4: Results of IROP catalyzed by NBu_4_SAc at 50 °C at various reaction times.^[a]^

| Entry | Time (min) | Residual **TCTn**(%)^[b]^ | P(**TCT**) (%)^[b]^ | **TCT** (%)^[b]^ | Sel. to P(**TCT**) (%)^[c]^ | M_n,GPC_  (kg mol^-1^) ^[d]^ | Đ^[e]^ | [M] (mol/L) | ln([M]_0_/[M]_t_) |
| --- | --- | --- | --- | --- | --- | --- | --- | --- | --- |
| 1 | 5 | 80 | 18 | 2 | 90 | 3.0 | 1.14 | 0.8 | 0.223 |
| 2 | 30 | 28 | 69 | 3 | 96 | 14.3 | 1.09 | 0.28 | 1.273 |
| 3 | 60 | 8 | 87 | 5 | 95 | 14.5 | 1.09 | 0.08 | 2.526 |
| 4 | 180 | 0 | 94 | 6 | 94 | 16.5 | 1.16 | 0 | - |

[a] For IROP conditions, see general procedure. Polymerizations were conducted at 50 °C. [b] Determined from the relative integrals in ^1^H-NMR. [c] Selectivity to polymer, with **TCT** as sole side product. [d] M_n,GPC_ determined by gel permeation chromatography in chloroform, calibrated with polystyrene standards; [e] Đ = M_w,GPC_/M_n,GPC_.

| Time (min) | ln([M]0/[M]) |
| --- | --- |
| 0 | 0 |
| 1 | 0.430783 |
| 5 | 1.96611 |
| 10 | 3.50656 |

| Time (min) | ln ([M]0/[M]) |
| --- | --- |
| 0 | 0 |
| 5 | 0.223144 |
| 30 | 1.27297 |
| 60 | 2.52573 |

Figure S3: Semilogarithmic plots of **TCTn** IROP catalyzed by NBu_4_SAc at 80 °C (left) and 50 °C (right).

## Polymer characterization

**^1^H-NMR** (400 MHz, CDCl_3_) δ 4.74 – 4.65 (m, 1H, H4), 3.21 – 3.04 (m, 1H, H1a), 2.79 – 2.55 (m, 1H, H1b), 2.55 – 2.35 (m, 1H, H2), 1.97 – 1.72 (m, 4H, H3a, H3b, H5a and H6a), 1.65 – 1.54 (m, 2H, H5b and H6b) ppm; **^13^C{^1^H}-NMR** (100 MHz, CDCl_3_) δ 199.1 – 198.7 (C8), 93.4 – 93.2 (C7), 77.7 (C4), 45.5 (C2), 45.3 (C2), 37.9 (C3), 34.7 (C5), 31.5 (C1 or C6), 31.3 (C1 or C6) ppm; **IR (ATR)**: ṽ = 2982 (w), 2874 (m), 1674 (s), 1450 (w) 1297 (w), 1193 (w), 1071 (w), 1048 (w), 1017 (w), 985 (w), 954 (w), 935 (w), 892 (m), 813 (m) cm^-1^.

**Example of conversion and selectivity determination with ^1^H-NMR.**

As there are no well-resolved signals for the polythioester, the total integration for the signals at 4.63 ppm (**H4** + **H1a**) and the signal at 3.25 ppm (**H1a**) is set to 100%. The relative ratios of starting material and products are then determined as follows:

- For the thionolactone: the integral of the signal at 4.12 ppm (**H1b**)
- For the polythioester: the integral of the signals at 4.63 ppm (**H4** + **H1a**) minus the integral of the signal at 4.12 ppm (**H1b**)
- For the thiolactone: the integral at 3.25 ppm (**H1a**)

Figure S4: Example of conversion and selectivity determination of IROP with ^1^H-NMR.

**Example of number average molecular weight (M_n,NMR_) determination**

The ratio of integrals in ^1^H-NMR between H2 at 2.45 ppm (see Figure S5) and the methyl group of the thioacetate initiator at 2.29 ppm are used to calculate the M_n,NMR_ according to M_n,NMR_ = n x monomer molecular weight + initiator molecular weight. An example is worked out in Figure S5 for P(**TCT**) formed according to Table S1, Entry 2, where n = 130 and M_n,NMR_ = 130 x 170.23 + 76.11 = 22206 g/mol.

Figure S5: Example of M_n,NMR_ determination for P(**TCT**).

## Ring-closing depolymerization

**In solid state**

Samples of polythioester of ~2 mg, (formed with [**TCTn**]/[^t^Bu-P_4_]/[BnOH] = 100/1/1, according to Table S1, Entry 1, containing 0.6 mol% residual ^t^Bu-P_4_) were placed in sealed aluminum DSC pans and heated from rt to 155 °C at a rate of 10 °C/min, and held at an isothermal for the times displayed in Table S5. Afterwards, the pan was opened and the polymer was dissolved in CDCl_3_ for ^1^H-NMR analysis to determine the ratio of P(**TCT**):**TCT**.

Table S5: Results of solid-state ring-closing depolymerization at 155 °C.

| Entry | Time kept at 155 °C (min) | P(**TCT**) (%) | **TCT** (%) |
| --- | --- | --- | --- |
| 1 | 0 | 96 | 4 |
| 2 | 10 | 78 | 23 |
| 3 | 20 | 55 | 45 |
| 4 | 30 | 39 | 61 |
| 5 | 60 | 22 | 78 |
| 6 | 90 | 0 | 100 |

**Optimization of base catalyzed RCD in solution**

*General procedure:* Polythioester (0.1-0.2 mmol, formed with [**TCTn**]/[NBu_4_SAc] = 100/1, according to Table S1, Entry 2) was dissolved in the solvent as indicated in Table S6 after which the indicated amount of catalyst was added and the reaction mixture was stirred at 20 °C or 100 °C. After the indicated time, the crude conversion and selectivity were determined with ^1^H-NMR analysis (see Table S6).

Using triethylamine (TEA) as catalyst resulted in low spectroscopic yields (<1 %) under the standard conditions (0.1 equiv. catalyst, 0.2 M in DCM at 20 °C,), as well as in more dilute conditions (0.1 equiv. TEA, 0.02 M in DCM at 100 °C), or with more catalyst (2 equiv. TEA, 0.2M in DCM at 20 °C).

Table S6: Results of optimization of base catalyzed ring-closing depolymerization.

| Entry | Cat. | Cat. equiv. | Solvent | T (°C) | Time (h) | Conversion (%) | Selectivity (%) | Thio yield (spectr.) (%) [a] |
| --- | --- | --- | --- | --- | --- | --- | --- | --- |
| 1 | ^t^Bu-P_4_ | 0.1 | Toluene | 20 | 22 | 21 | >99 | 21 |
| 2 | DBU | 0.1 | DCM | 20 | 24 | 45 | 89 | 40 |
| 3 | DBU | 0.1 | DCM | 100 | 24 | 18 | >99 | 18 |
| 4 | DBU | 0.1 | Toluene | 20 | 24 | 18 | 89 | 16 |
| 5 | DBU | 0.5 | THF | 20 | 22 | 31 | 84 | 26 |
| 6 | DBU | 0.5 | DCM | 20 | 24 | 42 | 83 | 35 |
| 7 | DBU | 2.0 | DCM | 20 | 24 | 63 | 79 | 50 |
| 8 | TBD | 0.1 | DCM | 20 | 24 | 73 | 92 | 67 |
| 9 | TBD | 0.5 | DCM | 20 | 24 | >99 | - | 77^[b]^ |

[a] Conversion, selectivity and spectroscopic yield were based on the relative integrals in ^1^H-NMR; [b] Performed at 0.7 mmol polymer scale and the isolated **TCT** yield was determined after purification with flash column chromatography.

**Reaction profile of RCD with DBU**

The depolymerization was done according to Table S6, Entry 6 (0.5 equiv. DBU), and samples were taken at the designated times and quenched with acetic acid. Conversion was determined with ^1^H-NMR analysis and the molecular weight of the residual polymer was determined with GPC (Table S7).

Table S7: Product distribution and GPC results of DBU catalyzed ring-closing depolymerization over time.

| Entry | Time (h) | P(**TCT**) | **TCT** | Side product (%) | M_n,GPC_ of total signal  (g/mol) | Đ  Total signal | M_n,GPC_ of single-chain peak (kg/mol) | Đ  of single-chain peak | M_p,GPC_ (kg/mol) |
| --- | --- | --- | --- | --- | --- | --- | --- | --- | --- |
| 1 | 0 | 99 | 1 | 0 | 15.5 | 1.11 | 14.8 | 1.06 | 16.1 |
| 2 | 1 | 97 | 3 | 0 | 16.6 | 1.14 | 15.2 | 1.05 | 16.2 |
| 3 | 2.25 | 95 | 5 | 0 | 16.3 | 1.17 | 15.1 | 1.06 | 16.3 |
| 4 | 5 | 92 | 8 | 0 | 16.8 | 1.11 | 15.6 | 1.04 | 16.3 |
| 5 | 24 | 58 | 35 | 8 | 13.2 | 1.15 | 13.2 | 1.15 | 16.0 |
| 6 | 96 | 35 | 47 | 17 | 10.7 | 1.22 | 10.7 | 1.22 | 15.7 |

Mp,GPC during depolymerization

| Polymer conversion (%) | Mp,GPC (kg/mol) |
| --- | --- |
| 0 | 16.097 |
| 3 | 16.231 |
| 5 | 16.322 |
| 8.5 | 16.324 |
| 42 | 15.97 |
| 65 | 15.674 |

Figure S6: The highest molecular weight peak (M_p_) remains ca. 16 kg/mol during RCD, indicating an unzipping length that is longer than the length of the polymers.

## Hydrolysis

**Hydrolysis of P(TCT) with 1 M NaOH.**

*Procedure:* The polymer (0.1 mmol) was suspended in 1 M NaOH in D_2_O (1 mL) and stirred at rt for 24 h. Analysis with ^1^H-NMR (D_2_O) shows **TCT-H_2_O***-2Na* and the oxidized product (**TCT-H_2_O**)_ox_ were formed in a 4:1 ratio (see Figure S37). After acidification of the aqueous layer with 4 M HCl until pH=1, the products were extracted with DCM (3 x ~ 10 mL) and the combined organic layers were dried over MgSO_4_ and evaporated *in vacuo* and analyzed with NMR, FTIR and HRMS (Figures S41-S46).

*Characterization* **TCT-H_2_O**-2Na*:***^1^H-NMR (**400 MHz, D_2_O) δ 4.51 (t, *J* = 5.0 Hz, 1H, H4), 2.57 (d, *J* = 9.1 Hz, 1H, H7), 2.06 – 1.93 (m, 2H, H7 and H6), 1.91 – 1.53 (m, 6H, H2, H3 and H5) ppm; **^13^C-NMR** (100 MHz, D_2_O) δ 178.9 (C8), 91.3 (C1), 76.7 (C4), 51.0 (C6), 37.6 (C2/3/5), 37.0 (C2/3/5), 30.0 (C2/3/5), 28.2 (C7) ppm.

*Characterization* **TCT-H_2_O***:***^1^H-NMR** (400 MHz, CDCl_­3_) δ 4.68 (t, *J* = 5.1 Hz, 1H, H4), 2.88 – 2.76 (m, 1H, H7), 2.38 – 2.19 (m, 2H), 2.02 – 1.77 (m, 4H, H2/3/5), 1.77 – 1.67 (m, 1H, H2/3/5), 1.65 – 1.56 (m, 1H, H2/3/5) ppm; **^13^C-NMR** (100 MHz, CDCl_3_) δ 174.3 (C8), 87.6 (C1), 77.5 (C4), 49.5 (C6), 37.7 (C2/3/5), 34.2 (C2/3/5), 30.6 (C2/3/5), 27.6 (C7) ppm. **IR (ATR)**: ṽ = 2956 (s), 2876 (m), 2561 (w, S-H stretch), 1730 (s), 1451 (w), 1415 (w), 1299 (w), 1259 (m), 1171 (w), 1116 (w), 1085 (w), 1048 (m), 1015 (w), 956 (w), 858 (w), 795 (w), 733 (w), 704 (w), 614 (w) cm^-1^. **ESI-MS:** *m/z* [*M-*H]^-^ calculated for C_8_H_11_O_3_S: 187.0429, found: 187.0429.

**Hydrolysis of P(TCT) with 4 M NaOH (followed over time)**

*Procedure*: Polythioester P(**TCT**) (26.0 mg, 0.153 mmol, 1.00 equiv.) was suspended in 4 M NaOH in D_2_O (1.11 mL, 4.43 mmol, 29 equiv.) and 3-(trimethylsilyl)-2,2,3,3-tetradeuteropropionic acid (TMSP-*d*_4_) (100 μL of 15 mg/mL solution in D_2_O, so 1.5 mg, 8.71 μmol, 0.057 equiv.) was added as internal standard. The suspension was stirred at rt and at the indicated times in Table S8, the suspension was transferred to an NMR tube and the formation of hydrolyzed product was measured with ^1^H-NMR (see Figure S7, S135 and separate ^1^H-NMR spectra on the YODA data repository).

Table S8: Product formation during hydrolysis of P(**TCT**), monitored by ^1^H-NMR.

| Entry | Time (h) | Conversion P(**TCT**) (%) | Spectroscopic yield of **TCT-H_2_O** (%) | Spectroscopic yield of (**TCT-H_2_O**)_ox_ (%) | [P(**TCT**)] (mol/L) | ln([P(**TCT**)]_0_/[P(**TCT**)]_t_ |
| --- | --- | --- | --- | --- | --- | --- |
| 1 | 17 | 11 | 11 | 0 | 0.122 | 0.120 |
| 2 | 26 | 24 | 23 | 0 | 0.105 | 0.273 |
| 3 | 42 | 43 | 42 | 1 | 0.079 | 0.558 |
| 4 | 51 | 54 | 50 | 4 | 0.063 | 0.777 |
| 5 | 66 | 66 | 62 | 4 | 0.047 | 1.076 |
| 6 | 90 | 81 | 74 | 8 | 0.026 | 1.677 |
| 7 | 160 | 90 | 76 | 14 | 0.013 | 2.333 |

Product distribution (%)

| Reaction time (h) | TCT-H2O | (TCT-H2O)ox | P(TCT) |
| --- | --- | --- | --- |
| 17 | 10.8608 | 0.454902 | 88.6843 |
| 26 | 23.4275 | 0.454902 | 76.1176 |
| 42 | 42.0784 | 0.682353 | 57.2392 |
| 51 | 50.4373 | 3.58235 | 45.9804 |
| 66 | 61.6961 | 4.20784 | 34.0961 |
| 90 | 73.8078 | 7.50588 | 18.6863 |
| 160 | 76.1961 | 14.102 | 9.70196 |

Figure S7: Product distribution plot of hydrolysis of P(**TCT**) with 4 M NaOH.

| Time (h) | lln([P]0/[P]t) |
| --- | --- |
| 17 | 0.120087 |
| 26 | 0.27289 |
| 42 | 0.557931 |
| 51 | 0.776955 |
| 66 | 1.07599 |
| 90 | 1.67738 |

Figure S8: Semilogarithmic plots of P(**TCT**) hydrolysis with 4M NaOH.

**Formation of** (**TCT-H_2_O**)_ox_

*Procedure*: Hydrolysis with 1 M NaOH in D_2_O resulted in ca 20% formation of (**TCT-H_2_O**)_ox_. Hydrolysis with 4 M NaOH in D_2_O results in full selectivity to the hydrolysis product in the early stages of the reaction. However, when left to react for longer, the oxidized product (**TCT-H_2_O**)_ox_ was formed up to 14% after 160 h.

The isolated **TCT-H_2_O** (after acidic work up) formed the oxidized product (**TCT-H_2_O**)_ox_ when left under an active air flow (Figure S47). The same product was formed when adding 1 equiv. of I_2_ to **TCT-H_2_O** (Figure S48), forming a mixture of (**TCT-H_2_O**)_ox_ and thiolactone **TCT**. The oxidized product could be isolated by adding 4 M NaOH, extracting **TCT** with DCM (3 x 10 mL), acidifying the aqueous layer with 4 M HCl, and extracting (**TCT-H_2_O**)_ox_ with DCM (3 x 10 mL), drying the organic layers over MgSO_4_ and concentration *in vacuo* (see Figure S49).

*Characterization* (**TCT-H_2_O**)_ox_*:***^1^H-NMR** (400 MHz, CDCl_3_) δ 4.64 (t, *J* = 5.0 Hz, 2H), 3.02 (dd, *J* = 11.9, 3.1 Hz, 2H), 2.94 (dd, *J* = 12.9, 3.0 Hz, 1H), 2.60 (ddt, *J* = 11.3, 7.7, 3.5 Hz, 1H), 2.45 (ddt, *J* = 11.4, 7.5, 3.6 Hz, 1H), 2.40 – 2.29 (m, 1H), 2.22 (dd, *J* = 12.9, 11.1 Hz, 1H), 1.99 – 1.79 (m, 9H), 1.73 – 1.62 (m, 2H), 1.58 (dt, *J* = 11.3, 6.5 Hz, 2H) ppm. **IR (ATR)**: ṽ = 2925 (s), 2954 (m), 1732 (m), 1453 (w), 1259 (w), 1112 (w), 1049 (w), 957 (w), 860 (w), 798 (w), 730 (w), 558 (w) cm^-1^. **ESI-MS:** *m/z* [*M*+H]^+^ calculated for C_16_H_23_O_6_S_2_: 375.0936, found: 375.0960, and *m/z* [*M*-H]^-^ calculated for C_16_H_21_O_6_S_2_: 373.077958 found: 373.0767.

**Hydrolysis of P(TCL) with 4 M NaOH (hydrolysis reaction profile)**

*Procedure*: The polyester P(**TCL**) (23.6 mg, 0.153 mmol, 1.00 equiv.) was suspended in 4 M NaOH in D_2_O (1.11 mL, 4.43 mmol, 29 equiv.) and 3-(trimethylsilyl)-2,2,3,3-tetradeuteropropionic acid (TMSP-*d*_4_) (100 μL of 15 mg/mL in D_2_O, so 1.5 mg, 8.71 μmol, 0.057 equiv.) was added as internal standard. The suspension was stirred at rt and at the indicated times in Table S9, the suspension was transferred to an NMR tube and the formation of hydrolyzed product was measured with ^1^H-NMR.

%

| hours | Procuts % | polymer % |
| --- | --- | --- |
| 0 | 0 | 100 |
| 1.83333 | 24.7635 | 75.2365 |
| 2.5 | 33.2653 | 66.7347 |
| 3.33333 | 42.7371 | 57.2629 |
| 4.66667 | 56.5453 | 43.4547 |
| 5.5 | 63.9059 | 36.0941 |
| 6.25 | 71.0382 | 28.9618 |
| 7.5 | 77.8853 | 22.1147 |
| 23.5 | 92.7206 | 7.27941 |

%

| hours | Procuts % | polymer % |
| --- | --- | --- |
| 0 | 0 | 100 |
| 1.83333 | 24.7635 | 75.2365 |
| 2.5 | 33.2653 | 66.7347 |
| 3.33333 | 42.7371 | 57.2629 |
| 4.66667 | 56.5453 | 43.4547 |
| 5.5 | 63.9059 | 36.0941 |
| 6.25 | 71.0382 | 28.9618 |
| 7.5 | 77.8853 | 22.1147 |
| 23.5 | 92.7206 | 7.27941 |

%

| hours | TCL-H2O | P(TCL) |
| --- | --- | --- |
| 0 | 0 | 100 |
| 1.83333 | 24.7635 | 75.2365 |
| 2.5 | 33.2653 | 66.7347 |
| 3.33333 | 42.7371 | 57.2629 |
| 4.66667 | 56.5453 | 43.4547 |
| 5.5 | 63.9059 | 36.0941 |
| 6.25 | 71.0382 | 28.9618 |
| 7.5 | 77.8853 | 22.1147 |
| 23.5 | 92.7206 | 7.27941 |

Table S9: Polymer conversion during hydrolysis of P(**TCL**) with 4 M NaOH, monitored by ^1^H-NMR.

| Entry | Time (h) | Conversion of P(**TCL**) (%) | [P] (mol/L) | ln([P]_0_/[P]_t_ |
| --- | --- | --- | --- | --- |
| 1 | 1h50 | 25 | 0.098 | 0.285 |
| 2 | 2h30 | 33 | 0.087 | 0.404 |
| 3 | 3h20 | 43 | 0.074 | 0.558 |
| 4 | 4h40 | 57 | 0.056 | 0.833 |
| 5 | 5h30 | 64 | 0.047 | 1.019 |
| 6 | 6h15 | 71 | 0.038 | 1.239 |
| 7 | 7h30 | 78 | 0.029 | 1.509 |
| 8 | 23h30 | 93 | 0.009 | 2.620 |

%

| hours | TCL-H2O | P(TCL) |
| --- | --- | --- |
| 0 | 0 | 100 |
| 1.83333 | 24.7635 | 75.2365 |
| 2.5 | 33.2653 | 66.7347 |
| 3.33333 | 42.7371 | 57.2629 |
| 4.66667 | 56.5453 | 43.4547 |
| 5.5 | 63.9059 | 36.0941 |
| 6.25 | 71.0382 | 28.9618 |
| 7.5 | 77.8853 | 22.1147 |
| 23.5 | 92.7206 | 7.27941 |

Product distribution (%)

| Reaction time (h) | TCL-H2O | P(TCL) |
| --- | --- | --- |
| 0 | 0 | 100 |
| 1.83333 | 24.7635 | 75.2365 |
| 2.5 | 33.2653 | 66.7347 |
| 3.33333 | 42.7371 | 57.2629 |
| 4.66667 | 56.5453 | 43.4547 |
| 5.5 | 63.9059 | 36.0941 |
| 6.25 | 71.0382 | 28.9618 |
| 7.5 | 77.8853 | 22.1147 |
| 23.5 | 92.7206 | 7.27941 |

Figure S9: Product distribution during hydrolysis of P(**TCL**) with 4 M NaOH.

| Reaction time (h) | ln([P]0/[P]t) |
| --- | --- |
| 1.83333 | 0.284534 |
| 2.5 | 0.404445 |
| 3.33333 | 0.557517 |
| 4.66667 | 0.833451 |
| 5.5 | 1.01904 |
| 6.25 | 1.23919 |
|  | 1.50893 |

Figure S10: Semilogarithmic plots of P(**TCL**) hydrolysis with 4M NaOH..

## Methanolysis

*Procedure:* P(**TCT**) (16.4 mg, 0.096 mmol) was dissolved in DCM (0.7 mL), and the solution was diluted with methanol (0.5 mL) (final concentration = 0.08 M). Solid K_2_CO_3_ (2.0 mg, 0.014 mmol, 0.15 equiv. w.r.t. to the polymer) was added and the solution was stirred at rt for 2 h. NMR analysis indicated partial conversion of the polymer and formation of **TCT-MeOH**, as well as the thiolactone **TCT** . Longer reaction time (24 h) led to a relative increase in **TCT-MeOH** production (Table S10). The reaction mixture was concentrated *in vacuo* and the crude was purified with by silica gel flash column chromatography (20 to 50 v/v% ethyl acetate in petroleum ether) to yield ~5 mg product (ca. 26% yield).

*Characterization* **TCT-MeOH**: **TLC** (ethyl acetate/petroleum ether, 75/25, v/v) R_f_ = 0.25; **^1^H-NMR** (400 MHz, CDCl_3_) δ 4.65 (t, *J* = 4.9 Hz, 1H, H4), 3.82 (d, *J* = 3.3 Hz, 3H, H9), 2.88 – 2.78 (m, 1H, H7a), 2.48 (ddd, *J* = 12.6, 7.0, 4.2 Hz, 1H, H7b), 2.35 (ddd, *J* = 12.4, 10.3, 5.8 Hz, 1H, H6), 1.98 – 1.79 (m, 4H), 1.72 – 1.62 (m, 1H), 1.62 – 1.55 (m, 1H) ppm; **ESI-MS:** *m/z* [*M-*H]^-^ calculated for C_9_H_13_O_3_S: 201.0585, found: 201.0582.

Table S10: Product distribution during methanolysis of P(**TCT**) as determined by ^1^H-NMR.

| Entry | Time (h) | Residual P(**TCT**) (%) | **TCT** (%) | **TCT-MeOH** (%) |
| --- | --- | --- | --- | --- |
| 1 | 2 | 19 | 34 | 48 |
| 2 | 24 | 17 | 15 | 68 |

**Methanolysis of P(TCT)**

*Procedure*: The polythioester P(**TCT**) (16.0 mg, 0.094 mmol, 1.00 equiv.) was dissolved in CDCl_3_ (0.564 mL), mesitylene as internal standard was added (ca 4 μL), and the solution was diluted with methanol-d_4_ (0.188 mL). A ^1^H-NMR spectrum was measured to determine the starting ratio of polymer/internal standard. Then 0.188 mL of a stock solution of potassium carbonate in methanol-d_4_ (0.025 M) was added (containing 0.0047 mmol K_2_CO_3_, 5 mol%). The final concentration of the polymer [P(**TCT**)]_0_ = 0.1 M, in a ratio of chloroform-d/methanol-d_4_ = 3 / 2. The reaction mixture was immediately transferred to the NMR tube and followed over time with ^1^H-NMR at 25 °C. The sample was shaken occasionally (after entry 4 and entry 9), which did not have an effect on the rate, indicating that the reaction is not diffusion-limited. Time measurement was started 2 min after adding the base, which in Table S11 has been added to the measured time intervals.

%

| hours | Procuts % | polymer % |
| --- | --- | --- |
| 0 | 0 | 100 |
| 1.83333 | 24.7635 | 75.2365 |
| 2.5 | 33.2653 | 66.7347 |
| 3.33333 | 42.7371 | 57.2629 |
| 4.66667 | 56.5453 | 43.4547 |
| 5.5 | 63.9059 | 36.0941 |
| 6.25 | 71.0382 | 28.9618 |
| 7.5 | 77.8853 | 22.1147 |
| 23.5 | 92.7206 | 7.27941 |

%

| hours | Procuts % | polymer % |
| --- | --- | --- |
| 0 | 0 | 100 |
| 1.83333 | 24.7635 | 75.2365 |
| 2.5 | 33.2653 | 66.7347 |
| 3.33333 | 42.7371 | 57.2629 |
| 4.66667 | 56.5453 | 43.4547 |
| 5.5 | 63.9059 | 36.0941 |
| 6.25 | 71.0382 | 28.9618 |
| 7.5 | 77.8853 | 22.1147 |
| 23.5 | 92.7206 | 7.27941 |

%

| hours | TCL-H2O | P(TCL) |
| --- | --- | --- |
| 0 | 0 | 100 |
| 1.83333 | 24.7635 | 75.2365 |
| 2.5 | 33.2653 | 66.7347 |
| 3.33333 | 42.7371 | 57.2629 |
| 4.66667 | 56.5453 | 43.4547 |
| 5.5 | 63.9059 | 36.0941 |
| 6.25 | 71.0382 | 28.9618 |
| 7.5 | 77.8853 | 22.1147 |
| 23.5 | 92.7206 | 7.27941 |

Table S11: Methanolysis of P(**TCT**) with 5 mol% K_2_CO_3_ monitored by ^1^H-NMR.

| Entry | Time | Residual P(**TCT**) (%) | **TCT** (%) | **TCT-MeOH** (%) | [P(**TCT**)] (mol/L) | ln([P(**TCT**)]_0_/[P(**TCT**)]_t_ |
| --- | --- | --- | --- | --- | --- | --- |
| 0 (start) | 0 | 100.0 | 0.0 | 0.0 | 0.1000 | 0.000 |
| 1 | 4 min 12 sec | 79.5 | 10.7 | 10.7 | 0.0795 | 0.229 |
| 2 | 5 min 16 sec | 75.9 | 12.9 | 12.9 | 0.0759 | 0.276 |
| 3 | 6 min 20 sec | 71.1 | 14.5 | 14.5 | 0.0711 | 0.341 |
| 4 | 7 min 26 sec | 68.7 | 16.5 | 16.5 | 0.0687 | 0.376 |
| 5 | 8 min 48 sec | 65.1 | 17.9 | 17.9 | 0.0651 | 0.430 |
| 6 | 9 min 54 sec | 60.2 | 19.5 | 19.5 | 0.0602 | 0.507 |
| 7 | 11 min 2 sec | 57.8 | 20.5 | 21.7 | 0.0578 | 0.548 |
| 8 | 12 min 13 sec | 55.4 | 22.4 | 23.5 | 0.0554 | 0.590 |
| 9 | 18 min | 43.4 | 26.5 | 30.1 | 0.0434 | 0.835 |
| 10 | 19 min 6 sec | 41.0 | 26.8 | 31.7 | 0.0410 | 0.892 |
| 11 | 33 min 18 sec | 27.7 | 29.8 | 42.9 | 0.0277 | 1.283 |
| 12 | 1 h 38 min | 7.2 | 25.3 | 67.5 | 0.0072 | 2.627 |

Product distribution (%)

| Time (min) | Polythioester (%) | TCT (%) | TCT-MeOH (%) |
| --- | --- | --- | --- |
| 0 | 100 | 0 | 0 |
| 4.2 | 79.5181 | 10.7143 | 10.7143 |
| 5.26667 | 75.9036 | 12.9412 | 12.9412 |
| 6.33333 | 71.0843 | 14.4578 | 14.4578 |
| 7.43333 | 68.6747 | 16.4706 | 16.4706 |
| 8.8 | 65.0602 | 17.8571 | 17.8571 |
| 9.9 | 60.241 | 19.5122 | 19.5122 |
| 11.0333 | 57.8313 | 20.4819 | 21.6867 |
| 12.2167 | 55.4217 | 22.3529 | 23.5294 |
| 18 | 43.3735 | 26.506 | 30.1205 |
| 19.1 | 40.9639 | 26.8293 | 31.7073 |
| 33.3 | 27.7108 | 29.7619 | 42.8571 |
| 98 | 7.22892 | 25.3012 | 67.4699 |

Figure S11: Product distribution during methanolysis of P(**TCT**) with 5 mol% K_2_CO_3_.

| Time (min) | ln([P]0/[P]t) |
| --- | --- |
| 4.2 | 0.229186 |
| 5.26667 | 0.275706 |
| 6.33333 | 0.341303 |
| 7.43333 | 0.375789 |
| 8.8 | 0.429857 |
| 9.9 | 0.506818 |
| 11.0333 | 0.54764 |
| 12.2167 | 0.590199 |
| 18 | 0.835322 |
| 19.1 | 0.89248 |

Figure S12: Semilogarithmic plots of P(**TCT**) methanolysis with 5 mol% K_2_CO_3_.

**Methanolysis of P(TCL)**

*Procedure*: The polyester P(**TCL**) (23.1 mg, 0.150 mmol, 1.00 equiv.) was dissolved in CDCl_3_ (0.9 mL), mesitylene as internal standard was added, and the solution was diluted with methanol-d_4_ (0.3 mL). A ^1^H-NMR spectrum was measured to determine the starting ratio of polymer/internal standard. Then 0.3 mL of a stock solution of potassium carbonate in methanol-d_4_ (0.025 M) was added (containing 0.0075 mmol K_2_CO_3_, 5 mol%). The final concentration of the polymer [P(**TCL**)]_0_ = 0.1 M, in a ratio of chloroform-d/methanol-d_4_ = 3 / 2. The sample was immediately transferred to the NMR tube and followed over time (see Table S12). The sample was occasionally taken out of the NMR and shaken (after entry 3, entry 6, entry 9, entry 12), which did not have an effect on the rate, indicating that the reaction is not diffusion-limited. The same procedure was followed for the methanolysis with 4 mol% K_2_CO_3_, with modified volumes of methanol-d_4_ and K_2_CO_3_ in methanol-d_4_ (see Table S13).

%

| hours | Procuts % | polymer % |
| --- | --- | --- |
| 0 | 0 | 100 |
| 1.83333 | 24.7635 | 75.2365 |
| 2.5 | 33.2653 | 66.7347 |
| 3.33333 | 42.7371 | 57.2629 |
| 4.66667 | 56.5453 | 43.4547 |
| 5.5 | 63.9059 | 36.0941 |
| 6.25 | 71.0382 | 28.9618 |
| 7.5 | 77.8853 | 22.1147 |
| 23.5 | 92.7206 | 7.27941 |

%

| hours | Procuts % | polymer % |
| --- | --- | --- |
| 0 | 0 | 100 |
| 1.83333 | 24.7635 | 75.2365 |
| 2.5 | 33.2653 | 66.7347 |
| 3.33333 | 42.7371 | 57.2629 |
| 4.66667 | 56.5453 | 43.4547 |
| 5.5 | 63.9059 | 36.0941 |
| 6.25 | 71.0382 | 28.9618 |
| 7.5 | 77.8853 | 22.1147 |
| 23.5 | 92.7206 | 7.27941 |

%

| hours | TCL-H2O | P(TCL) |
| --- | --- | --- |
| 0 | 0 | 100 |
| 1.83333 | 24.7635 | 75.2365 |
| 2.5 | 33.2653 | 66.7347 |
| 3.33333 | 42.7371 | 57.2629 |
| 4.66667 | 56.5453 | 43.4547 |
| 5.5 | 63.9059 | 36.0941 |
| 6.25 | 71.0382 | 28.9618 |
| 7.5 | 77.8853 | 22.1147 |
| 23.5 | 92.7206 | 7.27941 |

Table S12: Methanolysis of P(**TCL**) with 5 mol% K_2_CO_3_ monitored by ^1^H-NMR.

| Entry | Time | Residual P(**TCL**) (%) | [P] (mol/L) | ln([P]_0_/[P]_t_ |
| --- | --- | --- | --- | --- |
| 0 (start) | 0 | 100.0 | 0.1000 | 0.000 |
| 1 | 1 min 36 sec | 92.3 | 0.0923 | 0.080 |
| 2 | 2 min 43 sec | 87.5 | 0.0875 | 0.134 |
| 3 | 3 min 48 sec | 81.7 | 0.0817 | 0.202 |
| 4 | 5 min 13 sec | 77.9 | 0.0779 | 0.250 |
| 5 | 6 min 20 sec | 70.2 | 0.0702 | 0.354 |
| 6 | 7 min 28 sec | 64.4 | 0.0644 | 0.440 |
| 7 | 8 min 48 sec | 60.6 | 0.0606 | 0.501 |
| 8 | 9 min 56 sec | 53.8 | 0.0538 | 0.619 |
| 9 | 11 min 4 sec | 50.0 | 0.0500 | 0.693 |
| 10 | 12 min 30 sec | 48.1 | 0.0481 | 0.732 |
| 11 | 13 min 38 sec | 43.3 | 0.0433 | 0.838 |
| 12 | 14 min 48 sec | 40.4 | 0.0404 | 0.907 |
| 13 | 16 min 12 sec | 37.5 | 0.0375 | 0.981 |
| 14 | 17 min 19 sec | 33.7 | 0.0337 | 1.089 |
| 15 | 4 h 34 min | 6.7 | 0.0067 | 2.698 |
| 16 | 5 h 59 min | 6.7 | 0.0067 | 2.698 |

Product distribution

| Time (min) | Polyester (%) | TCL-MeOH (%) |
| --- | --- | --- |
| 0 | 100 | 1.88679 |
| 1.6 | 92.3077 | 9.43396 |
| 2.71667 | 87.5 | 15.0943 |
| 3.8 | 81.7308 | 20.7547 |
| 5.21667 | 77.8846 | 28.3019 |
| 6.33333 | 70.1923 | 32.0755 |
| 7.46667 | 64.4231 | 37.7358 |
| 8.8 | 60.5769 | 43.3962 |
| 9.93333 | 53.8462 | 47.1698 |
| 11.0667 | 50 | 50.9434 |
| 12.5 | 48.0769 | 56.6038 |
| 13.6333 | 43.2692 | 58.4906 |
| 14.8 | 40.3846 | 61.3208 |
| 16.2 | 37.5 | 66.9811 |
| 17.3167 | 33.6538 | 67.9245 |
| 274 | 6.73077 | 88.6792 |
| 359 | 6.73077 | 88.6792 |

Figure S13: Product distribution during methanolysis of P(**TCL**) with 5 mol% K_2_CO_3_.

| Time (min) | ln([P]0/[P]t) |
| --- | --- |
| 1.6 | 0.0800427 |
| 2.71667 | 0.133531 |
| 3.8 | 0.20174 |
| 5.21667 | 0.249942 |
| 6.33333 | 0.353931 |
| 7.46667 | 0.439698 |
| 8.8 | 0.501256 |
| 9.93333 | 0.619039 |
| 11.0667 | 0.693147 |
| 12.5 | 0.732368 |
| 13.6333 | 0.837728 |
| 14.8 | 0.906721 |
| 16.2 | 0.980829 |
| 17.3167 | 1.08904 |

Figure S14: Semilogarithmic plots of P(**TCL**) methanolysis with 5 mol% K_2_CO_3_.

Table S13: Methanolysis of P(**TCL**) with 4 mol% K_2_CO_3_ monitored by ^1^H-NMR.

| Entry | Time | Residual P(**TCL**) (%) | [P] (mol/L) | ln([P]_0_/[P]_t_ |
| --- | --- | --- | --- | --- |
| 0 (start) | 0 | 100.0 | 0.1000 | 0.000 |
| 1 | 4 min 11 sec | 95.4 | 0.0954 | 0.047 |
| 2 | 5 min 16 sec | 93.1 | 0.0931 | 0.071 |
| 3 | 6 min 23 sec | 92.0 | 0.0920 | 0.084 |
| 4 | 7 min 28 sec | 88.5 | 0.0885 | 0.122 |
| 5 | 8 min 22 sec | 86.2 | 0.0862 | 0.148 |
| 6 | 9 min 28 sec | 83.9 | 0.0839 | 0.175 |
| 7 | 10 min 58 sec | 83.9 | 0.0839 | 0.175 |
| 8 | 12 min 3 sec | 78.2 | 0.0782 | 0.246 |
| 9 | 13 min | 77.0 | 0.0770 | 0.261 |
| 10 | 54 min | 40.2 | 0.0402 | 0.911 |

Product distribution (%)

| Time (min) | Polyester (%) | TCL-MeOH (%) |
| --- | --- | --- |
| 0 | 100 | 1.88679 |
| 4.18333 | 95.4023 | 10.3774 |
| 5.26667 | 93.1034 | 12.2642 |
| 6.38333 | 91.954 | 15.0943 |
| 7.46667 | 88.5057 | 16.9811 |
| 8.36667 | 86.2069 | 18.8679 |
| 9.46667 | 83.908 | 21.6981 |
| 10.9667 | 83.908 | 23.5849 |
| 12.05 | 78.1609 | 24.5283 |
| 13 | 77.0115 | 26.4151 |
| 54 | 40.2299 | 54.717 |

Figure S15: Product distribution during methanolysis of P(**TCL**) with 4 mol% K_2_CO_3_.

| Time (min) | ln([P]0/[P]t) |
| --- | --- |
| 4.18333 | 0.0470675 |
| 5.26667 | 0.071459 |
| 6.38333 | 0.0838815 |
| 7.46667 | 0.122103 |
| 8.36667 | 0.14842 |
| 9.46667 | 0.175449 |
| 10.9667 | 0.175449 |
| 12.05 | 0.2464 |
| 13 | 0.261215 |

Figure S16: Semilogarithmic plots of P(**TCL**) methanolysis with 4 mol% K_2_CO_3_.

**Methanolysis competition experiment P(TCL) and P(TCT)**

*Procedure*: A 0.1 molar solution for both polymers was attempted: A mixture of polythioester P(**TCT**) (11.8 mg, 0.069 mmol, 1.00 equiv.) and polyester P(**TCL**) (9.9 mg, 0.064 mmol, 0.93 equiv.) was dissolved in CDCl_3_ (0.416 mL) and mesitylene as internal standard was added (ca 3 μL). The solution was diluted with methanol-d_4_ (0.139 mL), which caused solids to precipitate. CDCl_3_ (0.1 mL) was added to dissolve the polymers again. A ^1^H-NMR spectrum was measured to determine the starting ratio of polymers/internal standard. Then 0.129 mL of a stock solution of potassium carbonate in methanol-d_4_ (0.025 M) was added (containing 0.0035 mmol K_2_CO_3_, 5 mol%). Again, precipitation was observed and another 0.1 mL of CDCl_3_ was added, resulting in a homogeneous sample. The final volume was 0.616 mL CDCl_3_ and 0.277 mL methanol-d_4_, resulting in an initial concentration of the polymer [P(**TCT**)]_0_ = 0.078 M and P(**TCL**)]_0_ = 0.072 M in a ratio of chloroform-d/methanol-d_4_ = 2.22 / 1. The reaction mixture was immediately transferred to the NMR tube and followed over time with ^1^H-NMR (see Table S14). Conversion was based on the relative integrals, using for P(**TCT**) the signal at 4.71-4.61 ppm (1H) and for P(**TCL**) the signal at 4.17-3.76 (corresponds to 2H).

%

| hours | Procuts % | polymer % |
| --- | --- | --- |
| 0 | 0 | 100 |
| 1.83333 | 24.7635 | 75.2365 |
| 2.5 | 33.2653 | 66.7347 |
| 3.33333 | 42.7371 | 57.2629 |
| 4.66667 | 56.5453 | 43.4547 |
| 5.5 | 63.9059 | 36.0941 |
| 6.25 | 71.0382 | 28.9618 |
| 7.5 | 77.8853 | 22.1147 |
| 23.5 | 92.7206 | 7.27941 |

%

| hours | Procuts % | polymer % |
| --- | --- | --- |
| 0 | 0 | 100 |
| 1.83333 | 24.7635 | 75.2365 |
| 2.5 | 33.2653 | 66.7347 |
| 3.33333 | 42.7371 | 57.2629 |
| 4.66667 | 56.5453 | 43.4547 |
| 5.5 | 63.9059 | 36.0941 |
| 6.25 | 71.0382 | 28.9618 |
| 7.5 | 77.8853 | 22.1147 |
| 23.5 | 92.7206 | 7.27941 |

%

| hours | TCL-H2O | P(TCL) |
| --- | --- | --- |
| 0 | 0 | 100 |
| 1.83333 | 24.7635 | 75.2365 |
| 2.5 | 33.2653 | 66.7347 |
| 3.33333 | 42.7371 | 57.2629 |
| 4.66667 | 56.5453 | 43.4547 |
| 5.5 | 63.9059 | 36.0941 |
| 6.25 | 71.0382 | 28.9618 |
| 7.5 | 77.8853 | 22.1147 |
| 23.5 | 92.7206 | 7.27941 |

Table S14: Methanolysis of a mixture of P(**TCL**) and P(**TCT**) with 5 mol% K_2_CO_3_ monitored by ^1^H-NMR.

| Entry | Time | Residual P(**TCT**) (%) | Residual P(**TCL**) (%) | [P(**TCT**)] (mol/L) | [P(**TCL**)] (mol/L) | 1/[P(**TCT**)] | 1/[P(**TCL**)] |
| --- | --- | --- | --- | --- | --- | --- | --- |
| 0 (start) | 0 | 100.0 | 100.0 | 0.0780 | 0.0720 | 12.82 | 13.89 |
| 1 | 2 min 48 sec | 70.4 | 96.8 | 0.0549 | 0.0697 | 18.22 | 14.35 |
| 2 | 3 min 52 sec | 63.2 | 96.6 | 0.0493 | 0.0696 | 20.27 | 14.37 |
| 3 | 4 min 56 sec | 57.5 | 96.6 | 0.0449 | 0.0695 | 22.28 | 14.38 |
| 4 | 6 min 1 sec | 53.0 | 96.0 | 0.0413 | 0.0691 | 24.19 | 14.47 |
| 5 | 7 min 24 sec | 49.1 | 97.7 | 0.0383 | 0.0703 | 26.12 | 14.22 |
| 6 | 8 min 29 sec | 44.7 | 95.7 | 0.0348 | 0.0689 | 28.71 | 14.52 |
| 7 | 9 min 34 sec | 41.7 | 95.4 | 0.0325 | 0.0687 | 30.73 | 14.56 |
| 8 | 10 min 39 sec | 38.7 | 95.5 | 0.0302 | 0.0687 | 33.12 | 14.55 |
| 9 | 12 min 11 sec | 36.3 | 95.5 | 0.0283 | 0.0687 | 35.35 | 14.55 |
| 10 | 13 min 25 sec | 33.7 | 94.8 | 0.0263 | 0.0683 | 38.09 | 14.65 |
| 11 | 15 min 10 sec | 31.9 | 94.0 | 0.0249 | 0.0676 | 40.22 | 14.78 |
| 12 | 16 min 43 sec | 28.5 | 94.8 | 0.0222 | 0.0682 | 45.01 | 14.66 |
| 13 | 18 min 33 sec | 26.2 | 93.7 | 0.0204 | 0.0674 | 48.93 | 14.83 |
| 14 | 4 h | 2.0 | 91.3 | 0.0016 | 0.0657 | 638.66 | 15.21 |

Residual polymer (%)

| Time (min) | P(TCT) | P(TCL) |
| --- | --- | --- |
| 0 | 100 | 100 |
| 2.8 | 70.3549 | 96.7668 |
| 3.86667 | 63.2334 | 96.6349 |
| 4.93333 | 57.5338 | 96.5929 |
| 6.01667 | 52.9932 | 95.963 |
| 7.4 | 49.074 | 97.6666 |
| 8.48333 | 44.6529 | 95.6631 |
| 9.56667 | 41.7135 | 95.4232 |
| 10.65 | 38.7143 | 95.4772 |
| 12.1833 | 36.2648 | 95.4592 |
| 13.4167 | 33.6599 | 94.8293 |
| 15.1667 | 31.8796 | 93.9536 |
| 16.7167 | 28.4861 | 94.7634 |
| 18.55 | 26.2038 | 93.6596 |
| 240 | 2.00741 | 91.3083 |

Figure S17: Product distribution during methanolysis of a mixture of P(**TCL**) and P(**TCT**) with 5 mol% K_2_CO_3_.

1/[P]

| Time (min) | P(TCT) | P(TCL) |
| --- | --- | --- |
| 0 | 12.8205 | 13.8889 |
| 2.8 | 18.2226 | 14.3529 |
| 3.86667 | 20.2749 | 14.3725 |
| 4.93333 | 22.2835 | 14.3788 |
| 6.01667 | 24.1928 | 14.4732 |
| 7.4 | 26.1249 | 14.2207 |
| 8.48333 | 28.7115 | 14.5185 |
| 9.56667 | 30.7347 | 14.555 |
| 10.65 | 33.1157 | 14.5468 |
| 12.1833 | 35.3525 | 14.5496 |
| 13.4167 | 38.0883 | 14.6462 |
| 15.1667 | 40.2155 | 14.7827 |
| 16.7167 | 45.0062 | 14.6564 |
| 18.55 | 48.9261 | 14.8291 |

Figure S18: Inverse concentration plot of P(**TCL**) and P(**TCT**) during competition methanolysis with 5 mol% K_2_CO_3_.

# Computational details

## Ring opening

Model reactions of the ring opening isomerization of **TCTn** and the ring opening of **TCT** were examined using DFT calculations. Optimization was done using the B3LYP functional with 6-31G(d) basis set as implemented in Gaussian 16 C.02 program and the single point energy calculations were carried out with 6-311+G(d,p) basis set.[9] Grimme’s D3 correction scheme with Becke-Johnson damping was used in all calculations.[10] All structures corresponding to local minima showed no imaginary frequencies. Reaction Gibbs free energies (Δ*G*_298K_) were computed using the results of the normal-mode analysis within the ideal gas approximation at a pressure of 1 atm and temperature of 298.15 K (see Table S15).

The DFT calculation output files (.log) containing optimized geometries, total energy, and related computational data are available in the online repository.

Table S15: Single point energies of the optimized structures.

| Structure | G (a.u.) | G (kcal mol^−1^) |
| --- | --- | --- |
| Thionolactone (**TCTn**) | -859.534668 | -539366 |
| Thiolactone (**TCT**) | -859.560663 | -539382 |
| Polythioester repeat unit | -1450.958584 | -910490 |
| Polythionoester repeat unit | -1450.909778 | -910460 |
| Methyl thioacetate | -591.404393 | -371112 |
| Methyl thionoacetate | -591.378978 | -371096 |

Figure S19: Overview reaction free energies of ring-opening.

## Methanolysis

Model reactions of the methanolysis of P(**TCT**) and P(**TCL**) were examined using DFT calculations. Optimization was done using the B3LYP functional with 6-31+G(d,p) basis set as implemented in Gaussian 16 C.02 program.[9] Grimme’s D3 correction scheme with Becke-Johnson damping was used in all calculations.[10] Nature of the stationary points was confirmed by the vibrational analysis carried out at the same level of theory. All structures corresponding to local minima showed no imaginary frequencies, while transition state (TS) structures were characterized by a single imaginary frequency corresponding to the expected reaction coordinate. Bulk solvent (methanol) effects were accounted for using the PCM solvation model.[11] Reaction Gibbs free energies (Δ*G*_298K_) were computed using the results of the normal-mode analysis within the ideal gas approximation at a pressure of 1 atm and temperature of 298.15 K (see Table S16). Attempts to optimize the tetrahedral intermediate for thioester methanolysis failed and collapsed to the ester and thiolate (complex product).

The DFT calculation output files (.log) containing optimized geometries, total energy, and related computational data are available in the online repository.

Figure S20: Plotted energies of methanolysis of the P(**TCT**) repeat unit

Figure S21: Plotted energies of methanolysis of the P(**TCL**) repeat unit.

Table S16: Computed energies for methanolysis of P(**TCT**) repeat unit.

| Structure | Imaginary frequencies | G (a.u.) | G (kcal mol^−1^) | ΔG  (kcal mol^−1^) |
| --- | --- | --- | --- | --- |
| **Start (calc. separate)** |  | **-1565.99326** | **-982676** | **0** |
| *Polythioester repeat unit* | 0 | -1450.776934 | -910376 |  |
| *MeO anion* | 0 | -115.216326 | -72299 |  |
| **Complex start** | **0** | **-1565.980159** | **-982667** | **8.2** |
| **TS1** | **1** | **-1565.970819** | **-982662** | **14.1** |
| **Complex product** | **0** | **-1566.006966** | **-982684** | **-8.6** |
| **Final (calc. separate)** |  | **-1566.030105** | **-982699** | **-23.1** |
| *Polyester/thioester repeat unit* | 0 | -1127.802226 | -707707 |  |
| *MeS anion* | 0 | -438.227879 | -274992 |  |

Table S17: Computed energies for methanolysis of P(**TCL**) repeat unit.

| Structure | Imaginary frequencies | G (a.u.) | G (kcal mol^−1^) | ΔG  (kcal mol^−1^) |
| --- | --- | --- | --- | --- |
| **Start (calc. separate)** |  | **-920.050139** | **-577340** | **0.0** |
| *Polyester repeat unit* | 0 | *-804.833813* | *-505041* |  |
| *MeO anion* | 0 | *-115.216326* | *-72299* |  |
| **Complex start** | **0** | **-920.035614** | **-577331** | **9.1** |
| **TS1** | **1** | **-920.029066** | **-577327** | **13.2** |
| **Intermediate** | **0** | **-920.032497** | **-577329** | **11.1** |
| **TS2** | **1** | **-920.023527** | **-577323** | **16.7** |
| **Complex product** | **0** | **-920.032624** | **-577329** | **11.0** |

# Spectra

## Monomeric species

Figure S22: ^1^H-NMR spectrum (CDCl_3_) of **TCTn**

Figure S23:^13^C{^1^H}-NMR spectrum (CDCl_3_) of **TCTn.**

Figure S24: ^1^H-^1^H COSY spectrum (CDCl_3_) of **TCTn.**

Figure S25: ^1^H-^13^C HSQC spectrum (CDCl_3_) of **TCTn.**

Figure S26: ^1^H-^13^C HMBC spectrum (CDCl_3_) of **TCTn.**

Figure S27: ATR-IR spectrum of **TCTn.**

Intensity (a.u.)

| Wavelength (nm) | P(ThioEVL) |
| --- | --- |
| 800 | 0.039991 |
| 799 | 0.039919 |
| 798 | 0.039834 |
| 797 | 0.040249 |
| 796 | 0.040018 |
| 795 | 0.039975 |
| 794 | 0.040162 |
| 793 | 0.039979 |
| 792 | 0.039915 |
| 791 | 0.040091 |
| 790 | 0.040066 |
| 789 | 0.039936 |
| 788 | 0.040034 |
| 787 | 0.039926 |
| 786 | 0.040066 |
| 785 | 0.040397 |
| 784 | 0.040081 |
| 783 | 0.039846 |
| 782 | 0.040093 |
| 781 | 0.040148 |
| 780 | 0.039932 |
| 779 | 0.040277 |
| 778 | 0.040166 |
| 777 | 0.039918 |
| 776 | 0.040156 |
| 775 | 0.040371 |
| 774 | 0.040293 |
| 773 | 0.040454 |
| 772 | 0.04016 |
| 771 | 0.040011 |
| 770 | 0.040083 |
| 769 | 0.040058 |
| 768 | 0.040107 |
| 767 | 0.040369 |
| 766 | 0.039966 |
| 765 | 0.039966 |
| 764 | 0.040192 |
| 763 | 0.04041 |
| 762 | 0.040318 |
| 761 | 0.040382 |
| 760 | 0.040067 |
| 759 | 0.039942 |
| 758 | 0.040174 |
| 757 | 0.040128 |
| 756 | 0.040307 |
| 755 | 0.040431 |
| 754 | 0.040125 |
| 753 | 0.040117 |
| 752 | 0.040286 |
| 751 | 0.040326 |
| 750 | 0.040318 |
| 749 | 0.040262 |
| 748 | 0.040075 |
| 747 | 0.039956 |
| 746 | 0.040412 |
| 745 | 0.04026 |
| 744 | 0.040208 |
| 743 | 0.04038 |
| 742 | 0.040259 |
| 741 | 0.040189 |
| 740 | 0.040359 |
| 739 | 0.040267 |
| 738 | 0.04023 |
| 737 | 0.040167 |
| 736 | 0.040148 |
| 735 | 0.040071 |
| 734 | 0.04019 |
| 733 | 0.040153 |
| 732 | 0.040248 |
| 731 | 0.040287 |
| 730 | 0.040177 |
| 729 | 0.040164 |
| 728 | 0.040204 |
| 727 | 0.040104 |
| 726 | 0.040083 |
| 725 | 0.040144 |
| 724 | 0.039998 |
| 723 | 0.039988 |
| 722 | 0.039974 |
| 721 | 0.040016 |
| 720 | 0.040092 |
| 719 | 0.040183 |
| 718 | 0.040075 |
| 717 | 0.04003 |
| 716 | 0.039976 |
| 715 | 0.040072 |
| 714 | 0.040017 |
| 713 | 0.040116 |
| 712 | 0.040155 |
| 711 | 0.040219 |
| 710 | 0.0402 |
| 709 | 0.040176 |
| 708 | 0.040266 |
| 707 | 0.040271 |
| 706 | 0.040266 |
| 705 | 0.040245 |
| 704 | 0.040168 |
| 703 | 0.040122 |
| 702 | 0.040133 |
| 701 | 0.040181 |
| 700 | 0.04018 |
| 699 | 0.040153 |
| 698 | 0.040144 |
| 697 | 0.040026 |
| 696 | 0.040139 |
| 695 | 0.040295 |
| 694 | 0.040183 |
| 693 | 0.040109 |
| 692 | 0.040129 |
| 691 | 0.04013 |
| 690 | 0.040137 |
| 689 | 0.040193 |
| 688 | 0.040293 |
| 687 | 0.040367 |
| 686 | 0.040485 |
| 685 | 0.040423 |
| 684 | 0.040488 |
| 683 | 0.040493 |
| 682 | 0.04026 |
| 681 | 0.040243 |
| 680 | 0.040485 |
| 679 | 0.040424 |
| 678 | 0.040371 |
| 677 | 0.040408 |
| 676 | 0.040296 |
| 675 | 0.040361 |
| 674 | 0.040472 |
| 673 | 0.040416 |
| 672 | 0.040337 |
| 671 | 0.040365 |
| 670 | 0.040277 |
| 669 | 0.040302 |
| 668 | 0.040424 |
| 667 | 0.040372 |
| 666 | 0.040287 |
| 665 | 0.04027 |
| 664 | 0.040235 |
| 663 | 0.040296 |
| 662 | 0.040447 |
| 661 | 0.040385 |
| 660 | 0.040274 |
| 659 | 0.040317 |
| 658 | 0.040302 |
| 657 | 0.04018 |
| 656 | 0.040245 |
| 655 | 0.040337 |
| 654 | 0.040241 |
| 653 | 0.040254 |
| 652 | 0.040336 |
| 651 | 0.040329 |
| 650 | 0.040416 |
| 649 | 0.040382 |
| 648 | 0.040205 |
| 647 | 0.040195 |
| 646 | 0.040163 |
| 645 | 0.04021 |
| 644 | 0.040248 |
| 643 | 0.040279 |
| 642 | 0.040273 |
| 641 | 0.040322 |
| 640 | 0.040256 |
| 639 | 0.040193 |
| 638 | 0.040169 |
| 637 | 0.040115 |
| 636 | 0.040094 |
| 635 | 0.040091 |
| 634 | 0.040086 |
| 633 | 0.040077 |
| 632 | 0.040087 |
| 631 | 0.040112 |
| 630 | 0.040148 |
| 629 | 0.04014 |
| 628 | 0.040007 |
| 627 | 0.040027 |
| 626 | 0.040016 |
| 625 | 0.039992 |
| 624 | 0.039959 |
| 623 | 0.040117 |
| 622 | 0.040088 |
| 621 | 0.039995 |
| 620 | 0.039971 |
| 619 | 0.040107 |
| 618 | 0.040086 |
| 617 | 0.040171 |
| 616 | 0.039989 |
| 615 | 0.039978 |
| 614 | 0.040066 |
| 613 | 0.040104 |
| 612 | 0.040117 |
| 611 | 0.040126 |
| 610 | 0.040089 |
| 609 | 0.040013 |
| 608 | 0.040081 |
| 607 | 0.040136 |
| 606 | 0.040035 |
| 605 | 0.040171 |
| 604 | 0.040046 |
| 603 | 0.03993 |
| 602 | 0.039896 |
| 601 | 0.040052 |

Figure S28: UV-Vis absorption spectrum of thionolactone **TCTn** in DCM (25 mM), λ_max_ = 399 nm, ε = 18.8 L mol^-1^ cm^-1^.

Figure S29: ^1^H-NMR spectrum (CDCl_3_) of **TCT.**

Figure S30: ^13^C{^1^H}-NMR spectrum (CDCl_3_) of **TCT.**

Figure S31: ^1^H-^1^H COSY spectrum (CDCl_3_) of **TCT.**

Figure S32: ^1^H-^13^C HSQC spectrum (CDCl_3_) of **TCT.**

Figure S33:^1^H-^13^C HMBC spectrum (CDCl_3_) of **TCT.**

Figure S34: ATR-IR spectrum of **TCT.**

Figure S35: TGA (green) and DTG (blue) curves of isolated thiolactone **TCT**; T_onset_ = 145.1 °C, T_5%_ = 118.2 °C, T_max_ = 174.9 °C.

UV-VIS

Intensity (a.u.)

| Wavelength (nm) | P(ThioEVL) |
| --- | --- |
| 800 | 0 |
| 799 | 0.001617 |
| 798 | 0.001601 |
| 797 | 0.001534 |
| 796 | 0.001624 |
| 795 | 0.001541 |
| 794 | 0.001596 |
| 793 | 0.001291 |
| 792 | 0.001421 |
| 791 | 0.001467 |
| 790 | 0.001341 |
| 789 | 0.001396 |
| 788 | 0.001489 |
| 787 | 0.001337 |
| 786 | 0.001475 |
| 785 | 0.001507 |
| 784 | 0.001428 |
| 783 | 0.001315 |
| 782 | 0.001483 |
| 781 | 0.001512 |
| 780 | 0.00153 |
| 779 | 0.001602 |
| 778 | 0.001793 |
| 777 | 0.001601 |
| 776 | 0.001534 |
| 775 | 0.001703 |
| 774 | 0.001809 |
| 773 | 0.001564 |
| 772 | 0.001755 |
| 771 | 0.001584 |
| 770 | 0.001356 |
| 769 | 0.001668 |
| 768 | 0.001707 |
| 767 | 0.001473 |
| 766 | 0.001642 |
| 765 | 0.001643 |
| 764 | 0.001474 |
| 763 | 0.001802 |
| 762 | 0.001761 |
| 761 | 0.001764 |
| 760 | 0.001884 |
| 759 | 0.001768 |
| 758 | 0.001826 |
| 757 | 0.001837 |
| 756 | 0.001848 |
| 755 | 0.001804 |
| 754 | 0.001951 |
| 753 | 0.00188 |
| 752 | 0.001719 |
| 751 | 0.001762 |
| 750 | 0.001846 |
| 749 | 0.001833 |
| 748 | 0.001846 |
| 747 | 0.00175 |
| 746 | 0.001787 |
| 745 | 0.001844 |
| 744 | 0.001842 |
| 743 | 0.001793 |
| 742 | 0.001912 |
| 741 | 0.001864 |
| 740 | 0.001831 |
| 739 | 0.001742 |
| 738 | 0.00155 |
| 737 | 0.001835 |
| 736 | 0.001921 |
| 735 | 0.001788 |
| 734 | 0.001818 |
| 733 | 0.001868 |
| 732 | 0.001725 |
| 731 | 0.001921 |
| 730 | 0.001995 |
| 729 | 0.001703 |
| 728 | 0.001697 |
| 727 | 0.001862 |
| 726 | 0.001816 |
| 725 | 0.001925 |
| 724 | 0.001873 |
| 723 | 0.001678 |
| 722 | 0.001595 |
| 721 | 0.00178 |
| 720 | 0.001814 |
| 719 | 0.001925 |
| 718 | 0.001948 |
| 717 | 0.001688 |
| 716 | 0.001666 |
| 715 | 0.001789 |
| 714 | 0.001902 |
| 713 | 0.001848 |
| 712 | 0.001865 |
| 711 | 0.001804 |
| 710 | 0.00172 |
| 709 | 0.001782 |
| 708 | 0.001934 |
| 707 | 0.00201 |
| 706 | 0.001973 |
| 705 | 0.001869 |
| 704 | 0.001857 |
| 703 | 0.001905 |
| 702 | 0.001978 |
| 701 | 0.002066 |
| 700 | 0.001968 |
| 699 | 0.001869 |
| 698 | 0.001762 |
| 697 | 0.00179 |
| 696 | 0.001951 |
| 695 | 0.001999 |
| 694 | 0.001802 |
| 693 | 0.001665 |
| 692 | 0.001739 |
| 691 | 0.001832 |
| 690 | 0.00189 |
| 689 | 0.002049 |
| 688 | 0.002005 |
| 687 | 0.001817 |
| 686 | 0.002017 |
| 685 | 0.001997 |
| 684 | 0.001813 |
| 683 | 0.001875 |
| 682 | 0.001964 |
| 681 | 0.001959 |
| 680 | 0.001964 |
| 679 | 0.002012 |
| 678 | 0.001937 |
| 677 | 0.001917 |
| 676 | 0.001958 |
| 675 | 0.001947 |
| 674 | 0.001976 |
| 673 | 0.002049 |
| 672 | 0.001948 |
| 671 | 0.00185 |
| 670 | 0.001942 |
| 669 | 0.001917 |
| 668 | 0.0019 |
| 667 | 0.001913 |
| 666 | 0.001872 |
| 665 | 0.001836 |
| 664 | 0.001925 |
| 663 | 0.001997 |
| 662 | 0.001894 |
| 661 | 0.001808 |
| 660 | 0.001841 |
| 659 | 0.001823 |
| 658 | 0.001848 |
| 657 | 0.001941 |
| 656 | 0.001878 |
| 655 | 0.001789 |
| 654 | 0.001939 |
| 653 | 0.002051 |
| 652 | 0.002063 |
| 651 | 0.002022 |
| 650 | 0.002144 |
| 649 | 0.002053 |
| 648 | 0.002129 |
| 647 | 0.002349 |
| 646 | 0.002315 |
| 645 | 0.00223 |
| 644 | 0.002222 |
| 643 | 0.002182 |
| 642 | 0.002215 |
| 641 | 0.00242 |
| 640 | 0.002523 |
| 639 | 0.002263 |
| 638 | 0.002227 |
| 637 | 0.002318 |
| 636 | 0.002287 |
| 635 | 0.002401 |
| 634 | 0.00236 |
| 633 | 0.002172 |
| 632 | 0.002209 |
| 631 | 0.002285 |
| 630 | 0.002338 |
| 629 | 0.002404 |
| 628 | 0.002489 |
| 627 | 0.002212 |
| 626 | 0.002135 |
| 625 | 0.002381 |
| 624 | 0.002477 |
| 623 | 0.002508 |
| 622 | 0.002605 |
| 621 | 0.002352 |
| 620 | 0.002399 |
| 619 | 0.002463 |
| 618 | 0.002513 |
| 617 | 0.002549 |
| 616 | 0.002575 |
| 615 | 0.002461 |
| 614 | 0.002435 |
| 613 | 0.002575 |
| 612 | 0.002794 |
| 611 | 0.002591 |
| 610 | 0.002724 |
| 609 | 0.002531 |
| 608 | 0.002395 |
| 607 | 0.002647 |
| 606 | 0.002685 |
| 605 | 0.00269 |
| 604 | 0.002702 |
| 603 | 0.002502 |
| 602 | 0.002603 |
| 601 | 0.002677 |

Figure S36: UV-Vis absorption spectrum of thiolactone **TCT** in DCM (25 mM).

Figure S37: ^1^H-NMR spectrum (D_2_O) of hydrolysis product **TCT-H_2_O-**2Na (1M NaOH in D_2_O).

Figure S38: ^13^C{^1^H}-NMR APT spectrum (D_2_O) of **TCT-H_2_O-**2Na (1M NaOH in D_2_O).

Figure S39: ^1^H-^1^H COSY spectrum (D_2_O) of **TCT-H_2_O-**2Na (1M NaOH in D_2_O).

Figure S40: ^1^H-^13^C ASAP-HMQC spectrum (D_2_O) of **TCT-H_2_O-**2Na (1M NaOH in D_2_O).

Figure S41: ^1^H-NMR spectrum (CDCl_3_) of **TCT-H_2_O** after acidic work-up.

Figure S42: ^13^C-NMR spectrum (CDCl_3_) of **TCT-H_2_O** after acidic work-up.

Figure S43: ^1^H-^1^H COSY spectrum (CDCl_3_) of **TCT-H_2_O** after acidic work-up.

Figure S44: ^1^H-^13^C ASAP-HMQC spectrum (CDCl_3_) of **TCT-H_2_O** after acidic work-up.

Figure S45: ^1^H-^13^C HMBC spectrum (CDCl_3_) of **TCT-H_2_O** after acidic work-up.

Figure S46: ATR-IR spectrum of **TCT-H_2_O** after acidic work-up, with S-H stretch indicative for the thiol at 2561 cm^-1^.

Figure S47: ^1^H-NMR spectrum (CDCl_3_) of (**TCT-H_2_O**)_ox_, formed by leaving a sample of hydrolysis product under an air flow.

Figure S48: ^1^H-NMR spectrum of formation of (**TCT-H_2_O**)_ox_ (and **TCT**) with iodine.

Figure S49: ^1^H-NMR spectrum of isolated (**TCT-H_2_O**)_ox_, after basic/acidic extraction.

Figure S50: ATR-IR spectrum of (**TCT-H_2_O**)_ox_.

Figure S51: ^1^H-NMR spectrum (CDCl_3_) of isolated **TCT-MeOH**.

## Polymer spectra

Figure S52: ^1^H-NMR spectrum (CDCl_3_) of low molecular weight P(**TCT**), obtained with **TCTn**/^t^Bu-P_4_/BnOH = 20/1/1 (Table S1, Entry S1), M_n,NMR_ = 7.5 kg/mol.

Figure S53: ^13^C{^1^H}-NMR spectrum (CDCl_3_) of P(**TCT**), obtained with **TCTn**/^t^Bu-P_4_/BnOH = 20/1/1 (Table S1, Entry S1).

Figure S54: ^1^H-^1^H COSY spectrum (CDCl_3_) of P(**TCT**), obtained with **TCTn**/^t^Bu-P_4_/BnOH = 20/1/1 (Table S1, Entry S1).

Figure S55: ^1^H-^13^C ASAP-HMQC spectrum (CDCl_3_) of P(**TCT**), obtained with **TCTn**/^t^Bu-P_4_/BnOH = 20/1/1 (Table S1, Entry S1).

Figure S56: ^1^H-^13^C HMBC spectrum (CDCl_3_) of P(**TCT**), obtained with **TCTn**/^t^Bu-P_4_/BnOH = 20/1/1 (Table S1, Entry S1).

Figure S57: GPC trace of P(**TCT**), obtained with **TCTn**/^t^Bu-P_4_/BnOH = 20/1/1 (Table S1, Entry S1); M_n_,_GPC_ = 9115 g/mol, M_w_,_GPC_ = 11467 g/mol, Đ = 1.26.

Figure S58: ^1^H-NMR spectrum (CDCl_3_) of P(**TCT**), obtained with **TCTn**/^t^Bu-P_4_/BnOH = 100/1/1 (Table S1, Entry 1). Contains 0.6 mol% residual ^t^Bu-P_4_ catalyst, which reduced thermal stability of P(**TCT**) (see RCD in solid state for depolymerization).

Figure S59: TGA (green) and DTG (blue) curves of P(**TCT**), obtained with **TCTn**/[^t^Bu-P_4_]/BnOH = 100/1/1 (Table S1, Entry 1), containing 0.6 mol% residual catalyst; T_onset_ = 192.1 °C, T_5%_ = 184.1 °C, T_max_ = 207.0 °C.

Figure S60: GPC trace of P(**TCT**), obtained with **TCTn**/^t^Bu-P_4_/BnOH = 100/1/1 (Table S1, Entry 1); M_n_,_GPC_ = 25002 g/mol, M_w_,_GPC_ = 30619 g/mol, Đ = 1.22.

Figure S61: GPC trace of P(**TCT**), obtained with **TCTn**/^t^Bu-P_4_/BnOH = 100/1/1 (Table S1, Entry 1); integrated as two peaks, area of high MW peak = 37.7%.

Figure S62: ^1^H-NMR spectrum (CDCl_3_) of P(**TCT**), obtained with **TCTn**/^t^Bu-P_4_/BnOH = 100/1/1 (Table S1, Entry S2). Contains 0.25 mol% residual ^t^Bu-P_4_ catalyst which reduced thermal stability of P(**TCT**).

Figure S63: ^1^H-NMR spectrum (CDCl_3_) of P(**TCT**), obtained with **TCTn**/^t^Bu-P_4_/BnOH = 100/1/1 (Table S1, Entry S2); precipitated two more times. No residual ^t^Bu-P_4_ catalyst observed.

Figure S64: Zoom of 1H-NMR spectra of **TCTn**/tBu-P_4_/BnOH = 100/1/1 (Table S1, Entry S2); effect of multiple precipitations on catalyst content.

Figure S65: GPC trace of P(**TCT**), obtained with **TCTn**/^t^Bu-P_4_/BnOH = 100/1/1 (Table S1, Entry S2); M_n_,_GPC_ = 25127 g/mol, M_w_,_GPC_ = 29455 g/mol, Đ = 1.17.

Figure S66: GPC trace of P(**TCT**), obtained with **TCTn**/^t^Bu-P_4_/BnOH = 100/1/1 (Table S1, Entry S2); integrated as two peaks, area of high MW peak = 20.0%.

Figure S67: TGA (green) and DTG (blue) curves of P(**TCT**), obtained with **TCTn**/[^t^Bu-P_4_]/BnOH = 100/1/1 (Table S1, Entry S2), containing 0.25 mol% residual catalyst; T_onset_ = 215.6 °C, T_5%_ = 167.6 °C, T_max_ = 234.3 °C

Figure S68: TGA (green) and DTG (blue) curves of P(**TCT**), obtained with **TCTn**/^t^Bu-P_4_/BnOH = 100/1/1, (Table S1, Entry S2) precipitated two more times; T_onset_ = 205.0 °C, T_5%_ = 209.2 °C, T_max_ = 213.4, 331.5 °C.

Figure S69: MDSC thermogram (second heating scan) of P(**TCT**), obtained with **TCTn**/^t^Bu-P_4_/BnOH = 100/1/1, (Table S1, Entry S2); T_g_ = 164.1 °C.

Figure S70: ^1^H-NMR spectrum (CDCl_3_) of P(**TCT**), obtained with **TCTn**/NBu_4_SAc= 100/1 (Table S1, Entry 2). n = 132, M_n,NMR_ = 22.2 kg/mol.

Figure S71: ^13^C{^1^H}-NMR spectrum (CDCl_3_) of P(**TCT**), obtained with **TCTn**/NBu_4_SAc= 100/1 (Table S1, Entry 2).

Figure S72: GPC trace of P(**TCT**), obtained with **TCTn**/NBu_4_SAc= 100/1 (Table S1, Entry 2); M_n_,_GPC_ = 21730 g/mol, M_w_,_GPC_ = 23392 g/mol, Đ = 1.08.

Figure S73: GPC trace of P(**TCT**), obtained with **TCTn**/NBu_4_SAc= 100/1 (Table S1, Entry 2); integrated as two peaks, area of high MW peak = 8.0%.

Figure S74: GPC trace of P(**TCT**), obtained with **TCTn**/NBu_4_SAc= 100/1 (Table S1, Entry 2) after 20 months in storage, integrated as two peaks, area of high MW peak = 7.9%.

Figure S75: TGA (green) and DTG (blue) curves of P(**TCT**), obtained with **TCTn**/NBu_4_SAc= 100/1 (Table S1, Entry 2); T_onset_ = 261.7 °C, T_5%_ = 255.7 °C, T_max_ = 294.7 °C, T_50%_ = 290.8 °C.

Figure S76: MDSC thermogram (second heating scan) of P(**TCT**), obtained with **TCTn**/NBu_4_SAc= 100/1 (Table S1, Entry 2); T_g_ = 167.3 °C

Figure S77: ^1^H-NMR spectrum (CDCl_3_) of isolated P(**TCT**), obtained with **TCTn**/NBu_4_SAc= 200/1 (Table S1, Entry 3); n = 211, M_n,NMR_ = 36.0 kg/mol.

Figure S78: Zoom of ^1^H-NMR spectrum (CDCl_3_) and peak assignment of isolated P(**TCT**), obtained with **TCTn**/NBu_4_SAc= 200/1 (Table S1, Entry 3).

Figure S79: ^13^C{^1^H}-NMR spectrum (CDCl_3_) of isolated P(**TCT**), obtained with **TCTn**/NBu4SAc= 200/1 (Table S1, Entry 3) and peak assignment.

Figure S80: ATR-FTIR spectrum of isolated P(**TCT**), obtained with **TCTn**/NBu_4_SAc= 200/1 (Table S1, Entry 3).

Figure S81: GPC trace of P(**TCT**), obtained with **TCTn**/NBu_4_SAc= 200/1 (Table S1, Entry 3); M_n_,_GPC_ = 37585 g/mol, M_w_,_GPC_ = 44142 g/mol, Đ = 1.17.

Figure S82: GPC trace of P(**TCT**), obtained with **TCTn**/NBu4SAc= 200/1 (Table S1, Entry 3); integrated as two peaks, area of high MW peak = 23.9 %.

Figure S83: TGA (green) and DTG (blue) curves of P(**TCT**), obtained with **TCTn**/NBu_4_SAc= 200/1 (Table S1, Entry 3); T_onset_ = 281.3 °C, T_5%_ = 272.7 °C, T_max_ = 313.7 °C and T_50%_ = 311.7 °C.

Figure S84: MDSC thermogram (second heating scan) of P(**TCT**), obtained with **TCTn**/NBu_4_SAc= 200/1 (Table S1, Entry 3); T_g_ = 169.4 °C.

Figure S85: ^1^H-NMR spectrum (CDCl_3_) of isolated P(**TCT**), obtained with **TCTn**/NBu_4_SAc= 400/1 (Table S1, Entry 4); n = 409, M_n,NMR_ = 69.7 kg/mol.

Figure S86: ^13^C{^1^H}-NMR spectrum (CDCl_3_) of isolated P(**TCT**), obtained with **TCTn**/NBu4SAc= 400/1 (Table S1, Entry 4).

Figure S87: ATR-FTIR spectrum of isolated P(**TCT**), obtained with **TCTn**/NBu_4_SAc= 400/1 (Table S1, Entry 4).

Figure S88: GPC trace of P(**TCT**), obtained with **TCTn**/NBu_4_SAc= 400/1 (Table S1, Entry 4); M_n_,_GPC_ = 64172 g/mol, M_w_,_GPC_ = 83787 g/mol, Đ = 1.31.

Figure S89: GPC trace of P(**TCT**), obtained with **TCTn**/NBu_4_SAc= 400/1 (Table S1, Entry 4); integrated as two peaks, area of high MW peak = 38.8%.

Figure S90: TGA (green) and DTG (blue) curves of P(**TCT**), obtained with **TCTn**/NBu_4_SAc= 400/1 (Table S1, Entry 4); T_onset_ = 279.9 °C, T_5%_ = 277.9 °C, T_max_ = 303.9 °C and T_50%_ = 333.5 °C.

Figure S91: MDSC thermogram (second heating scan) of P(**TCT**), obtained with **TCTn**/NBu_4_SAc= 4­­00/1 (Table S1, Entry 4); T_g_ = 171.3 °C.

Figure S92: ^1^H-NMR spectrum (CDCl_3_) of isolated P(**TCT**), obtained with **TCTn**/NBu_4_SAc= 600/1 (Table S1, Entry 5); n = 606, M_n,NMR_ = 103.2 kg/mol.

Figure S93: ^13^C{^1^H}-NMR spectrum (CDCl_3_) of isolated P(**TCT**), obtained with **TCTn**/NBu4SAc= 600/1 (Table S1, Entry 5).

Figure S94: ATR-FTIR spectrum of isolated P(**TCT**), obtained with **TCTn**/NBu_4_SAc= 600/1 (Table S1, Entry 5).

Intesnity (a.u.)

| Wavelength (nm) | P(ThioEVL) |
| --- | --- |
| 800 | 0.007568 |
| 799 | 0.00765 |
| 798 | 0.007705 |
| 797 | 0.008096 |
| 796 | 0.007889 |
| 795 | 0.007962 |
| 794 | 0.008035 |
| 793 | 0.007603 |
| 792 | 0.007602 |
| 791 | 0.007639 |
| 790 | 0.007523 |
| 789 | 0.007389 |
| 788 | 0.007432 |
| 787 | 0.007276 |
| 786 | 0.007245 |
| 785 | 0.007576 |
| 784 | 0.007247 |
| 783 | 0.006888 |
| 782 | 0.007121 |
| 781 | 0.007411 |
| 780 | 0.007124 |
| 779 | 0.007373 |
| 778 | 0.007184 |
| 777 | 0.006931 |
| 776 | 0.007166 |
| 775 | 0.007321 |
| 774 | 0.00732 |
| 773 | 0.007336 |
| 772 | 0.007281 |
| 771 | 0.007062 |
| 770 | 0.007377 |
| 769 | 0.007231 |
| 768 | 0.007353 |
| 767 | 0.007547 |
| 766 | 0.007301 |
| 765 | 0.007252 |
| 764 | 0.007375 |
| 763 | 0.007436 |
| 762 | 0.007389 |
| 761 | 0.007632 |
| 760 | 0.007434 |
| 759 | 0.007184 |
| 758 | 0.007261 |
| 757 | 0.007357 |
| 756 | 0.007475 |
| 755 | 0.007641 |
| 754 | 0.00749 |
| 753 | 0.007369 |
| 752 | 0.007516 |
| 751 | 0.007622 |
| 750 | 0.007725 |
| 749 | 0.007681 |
| 748 | 0.00757 |
| 747 | 0.007426 |
| 746 | 0.0077 |
| 745 | 0.007756 |
| 744 | 0.007811 |
| 743 | 0.007902 |
| 742 | 0.007806 |
| 741 | 0.00782 |
| 740 | 0.008102 |
| 739 | 0.00803 |
| 738 | 0.007949 |
| 737 | 0.008036 |
| 736 | 0.007963 |
| 735 | 0.007959 |
| 734 | 0.008142 |
| 733 | 0.0081 |
| 732 | 0.008201 |
| 731 | 0.008271 |
| 730 | 0.008126 |
| 729 | 0.008115 |
| 728 | 0.008206 |
| 727 | 0.008084 |
| 726 | 0.008121 |
| 725 | 0.008185 |
| 724 | 0.00799 |
| 723 | 0.00789 |
| 722 | 0.008056 |
| 721 | 0.008019 |
| 720 | 0.008206 |
| 719 | 0.008234 |
| 718 | 0.007935 |
| 717 | 0.007955 |
| 716 | 0.008215 |
| 715 | 0.008211 |
| 714 | 0.008131 |
| 713 | 0.008274 |
| 712 | 0.008251 |
| 711 | 0.008218 |
| 710 | 0.008321 |
| 709 | 0.008358 |
| 708 | 0.008344 |
| 707 | 0.008555 |
| 706 | 0.008594 |
| 705 | 0.008596 |
| 704 | 0.008655 |
| 703 | 0.008652 |
| 702 | 0.008466 |
| 701 | 0.00861 |
| 700 | 0.008682 |
| 699 | 0.008664 |
| 698 | 0.008642 |
| 697 | 0.008557 |
| 696 | 0.008496 |
| 695 | 0.008652 |
| 694 | 0.008666 |
| 693 | 0.008496 |
| 692 | 0.008425 |
| 691 | 0.008438 |
| 690 | 0.008431 |
| 689 | 0.008448 |
| 688 | 0.008476 |
| 687 | 0.008603 |
| 686 | 0.008641 |
| 685 | 0.008718 |
| 684 | 0.008798 |
| 683 | 0.008792 |
| 682 | 0.008732 |
| 681 | 0.008668 |
| 680 | 0.008757 |
| 679 | 0.008734 |
| 678 | 0.008783 |
| 677 | 0.008886 |
| 676 | 0.008871 |
| 675 | 0.008921 |
| 674 | 0.009017 |
| 673 | 0.00907 |
| 672 | 0.00914 |
| 671 | 0.009109 |
| 670 | 0.009001 |
| 669 | 0.009069 |
| 668 | 0.009254 |
| 667 | 0.009364 |
| 666 | 0.009342 |
| 665 | 0.009237 |
| 664 | 0.009207 |
| 663 | 0.009321 |
| 662 | 0.009405 |
| 661 | 0.009318 |
| 660 | 0.00926 |
| 659 | 0.009292 |
| 658 | 0.009295 |
| 657 | 0.009298 |
| 656 | 0.009298 |
| 655 | 0.009295 |
| 654 | 0.009341 |
| 653 | 0.009307 |
| 652 | 0.009289 |
| 651 | 0.009326 |
| 650 | 0.009403 |
| 649 | 0.009374 |
| 648 | 0.009334 |
| 647 | 0.009417 |
| 646 | 0.009328 |
| 645 | 0.00948 |
| 644 | 0.009691 |
| 643 | 0.009772 |
| 642 | 0.009872 |
| 641 | 0.009981 |
| 640 | 0.010017 |
| 639 | 0.010099 |
| 638 | 0.010261 |
| 637 | 0.010314 |
| 636 | 0.01037 |
| 635 | 0.010538 |
| 634 | 0.010517 |
| 633 | 0.010484 |
| 632 | 0.010637 |
| 631 | 0.010695 |
| 630 | 0.010659 |
| 629 | 0.01078 |
| 628 | 0.010815 |
| 627 | 0.010852 |
| 626 | 0.010903 |
| 625 | 0.010917 |
| 624 | 0.010699 |
| 623 | 0.010683 |
| 622 | 0.0106 |
| 621 | 0.010505 |
| 620 | 0.010545 |
| 619 | 0.010586 |
| 618 | 0.01056 |
| 617 | 0.01064 |
| 616 | 0.010577 |
| 615 | 0.010463 |
| 614 | 0.010539 |
| 613 | 0.010549 |
| 612 | 0.010513 |
| 611 | 0.010485 |
| 610 | 0.010597 |
| 609 | 0.010611 |
| 608 | 0.010686 |
| 607 | 0.010867 |
| 606 | 0.010802 |
| 605 | 0.01078 |
| 604 | 0.010889 |
| 603 | 0.010747 |
| 602 | 0.01066 |
| 601 | 0.010887 |

Figure S95: UV-Vis absorption spectrum of polythioester P(**TCT**) in DCM (25 mM), obtained with **TCTn**/NBu_4_SAc= 600/1 (Table S1, Entry 5).

Figure S96: GPC trace of P(**TCT**), obtained with **TCTn**/NBu_4_SAc= 600/1 (Table S1, Entry 5); M_n_,_GPC_ = 75274 g/mol, M_w_,_GPC_ = 101131 g/mol, Đ = 1.34.

Figure S97: GPC trace of P(**TCT**), obtained with **TCTn**/NBu_4_SAc= 600/1 (Table S1, Entry 5); integrated as two peaks, area of high MW peak = 36.6%.

Figure S98: GPC trace of P(**TCT**), obtained with **TCTn**/NBu_4_SAc= 600/1 (Table S1, Entry 5); after 16 months in storage; integrated as two peaks, area of high MW peak = 35.2%.

Figure S99: TGA (green) and DTG (blue) curves of P(**TCT**), obtained with **TCTn**/NBu_4_SAc= 600/1 (Table S1, Entry 5); T_onset_ = 280.0 °C, T_5%_ = 261.2 °C, T_max_ = 309.0 °C and T_50%_ = 318.6 °C.

Figure S100: MDSC thermogram (second heating scan) of P(**TCT**), obtained with **TCTn**/NBu_4_SAc= 600/1 (Table S1, Entry 5); T_g_ = 171.7 °C.

Figure S101: ^1^H-NMR spectrum (CDCl_3_) of isolated P(**TCT**), obtained with **TCTn**/NBu_4_SAc= 800/1 (Table S1, Entry 6); n = 772, M_n,NMR_ = 131.5 kg/mol.

Figure S102: ^13^C{^1^H}-NMR spectrum (CDCl_3_) of isolated P(**TCT**), obtained with **TCTn**/NBu_4_SAc= 800/1 (Table S1, Entry 6).

Figure S103: ATR-FTIR spectrum of isolated P(**TCT**), obtained with **TCTn**/NBu_4_SAc= 800/1 (Table S1, Entry 6).

a

Figure S104: GPC trace of P(**TCT**), obtained with **TCTn**/NBu_4_SAc= 800/1 (Table S1, Entry 6); M_n_,_GPC_ = 91861 g/mol, M_w_,_GPC_ = 127711 g/mol, Đ = 1.39.

Figure S105: GPC trace of P(**TCT**), obtained with **TCTn**/NBu_4_SAc= 800/1 (Table S1, Entry 6); integrated as two peaks, area of high MW peak = 48.0%.

Figure S106: TGA (green) and DTG (blue) curves of P(**TCT**), obtained with **TCTn**/NBu_4_SAc= 800/1 (Table S1, Entry 6); T_onset_ = 283.0 °C, T_5%_ = 272.0 °C, T_max_ = 305.1 °C and T_50%_ = 309.3 °C.

Figure S107: MDSC thermogram (second heating scan) of P(**TCT**), obtained with **TCTn**/NBu_4_SAc= 8­­00/1 (Table S1, Entry 6); T_g_ = 171.3 °C.

## **Spectra - polymerization profile**

IROP catalyzed by ^t^Bu-P_4_

Figure S108: Crude ^1^H-NMR spectrum (CDCl_3_) of IROP with ^t^Bu-P_4_ after the indicated times (Table S2). Thionolactone (blue), polythioester (green) and thiolactone (orange).

Figure S109: GPC trace of P(**TCT**), obtained with ^t^Bu-P_4_ after 30 sec (Table S2, Entry 1); M_n_,_GPC_ = 21.1 kg/mol, M_w_,_GPC_ = 22.6 kg/mol, Đ = 1.07.

Figure S110: GPC trace of P(**TCT**), obtained with ^t^Bu-P_4_ after 1 min (Table S2, Entry 2); integrated as one peak, M_n_,_GPC_ = 27.9 kg/mol, M_w_,_GPC_ = 31.0 kg/mol, Đ = 1.11.

Figure S111: GPC trace of P(**TCT**), obtained with ^t^Bu-P_4_ after 1 min (Table S2, Entry 2); integrated as two peaks, area of high MW peak = 11%

Figure S112: GPC trace of P(**TCT**), obtained with ^t^Bu-P_4_ after 10 min (Table S2, Entry 3); integrated as one peak, M_n_,_GPC_ = 25.1 kg/mol, M_w_,_GPC_ = 29.8 kg/mol, Đ = 1.19.

Figure S113: GPC trace of P(**TCT**), obtained with ^t^Bu-P_4_ after 10 min (Table S2, Entry 3); integrated as two peaks, area of high MW peak = 32%

Figure S114: GPC trace of P(**TCT**), obtained with ^t^Bu-P_4_ after 2 h (Table S2, Entry 4); integrated as one peak, M_n_,_GPC_ = 33.9 kg/mol, M_w_,_GPC_ = 42.2 kg/mol, Đ = 1.24.

Figure S115: GPC trace of P(**TCT**), obtained with ^t^Bu-P_4_ after 2 h (Table S2, Entry 4); integrated as two peaks, area of high MW peak = 14%

Figure S116: GPC trace of P(**TCT**), obtained with ^t^Bu-P_4_ after 21 h (Table S2, Entry 5); integrated as two peaks, area of high MW peak = 12%

Figure S117: GPC trace of P(**TCT**), obtained with ^t^Bu-P_4_ after 46 h (Table S2, Entry 6); only a minor amount of low molecular weight polymer is left.

Polymerization catalyzed by NBu_4_SAc at 80 °C

Figure S118: Crude ^1^H-NMR spectrum (CDCl_3_) of IROP with NBu_4_SAc at 80 °C at the indicated times (Table S3). Thionolactone (blue), polythioester (green) and thiolactone (orange).

Figure S119: GPC trace of P(**TCT**), obtained with NBu_4_SAc at 80 ^°^C after 1 min (Table S3, Entry 1); integrated as one peak, M_n_,_GPC_ = 5.9 kg/mol, M_w_,_GPC_ = 6.5 kg/mol, Đ = 1.10.

Figure S120: GPC trace of P(**TCT**), obtained with NBu_4_SAc at 80 ^°^C after 1 min (Table S3, Entry 1); integrated as two peaks, area of high MW peak = 5%

Figure S121: GPC trace of P(**TCT**), obtained with NBu_4_SAc at 80 °C after 5 min (Table S3, Entry 2); integrated as one peak, M_n_,_GPC_ = 11.6 kg/mol, M_w_,_GPC_ = 12.6 kg/mol, Đ = 1.09.

Figure S122: GPC trace of **P(TCT)**, obtained with NBu_4_SAc at 80 °C after 5 min (Table S3, Entry 2); integrated as two peaks, area of high MW peak = 6%.

Figure S123: GPC trace of P(**TCT**), obtained with NBu_4_SAc at 80 °C after 10 min (Table S3, Entry 3); integrated as one peak, M_n_,_GPC_ = 16.6 kg/mol, M_w_,_GPC_ = 18.4 kg/mol, Đ = 1.11.

Figure S124: GPC trace of P(**TCT**), obtained with NBu_4_SAc at 80 °C after 10 min (Table S3, Entry 3); integrated as two peaks, area of high MW peak = 9%

Figure S125: GPC trace of P(**TCT**), obtained with NBu_4_SAc at 80 °C after 30 min (Table S3, Entry 4); integrated as one peak, M_n_,_GPC_ = 16.0 kg/mol, M_w_,_GPC_ = 17.4 kg/mol, Đ = 1.14.

Figure S126: GPC trace of P(**TCT**), obtained with NBu_4_SAc at 80 °C after 30 min (Table S3, Entry 4); integrated as two peaks, area of high MW peak = 8%.

Polymerization catalyzed by NBu_4_SAc at 50 °C

Figure S127: Crude ^1^H-NMR spectrum (CDCl_3_) of IROP with NBu_4_SAc at 50 °C after the indicated times (Table S4). Thionolactone (blue), polythioester (green) and thiolactone (orange).

Figure S128: GPC trace of P(**TCT**), obtained with NBu_4_SAc at 50 °C after 5 min (Table S4, Entry 1); integrated as one peak, M_n_,_GPC_ = 3.0 kg/mol, M_w_,_GPC_ = 3.5 kg/mol, Đ = 1.14.

Figure S129: GPC trace of P(**TCT**), obtained with NBu_4_SAc at 50 °C after 30 min (Table S4, Entry 2); integrated as one peak, M_n_,_GPC_ = 14.3 kg/mol, M_w_,_GPC_ = 15.7 kg/mol, Đ = 1.09.

Figure S130: GPC trace of P(**TCT**), obtained with NBu_4_SAc at 50 °C after 60 min (Table S4, Entry 3); integrated as one peak, M_n_,_GPC_ = 14.5 kg/mol, M_w_,_GPC_ = 15.8 kg/mol, Đ = 1.09.

Figure S131: GPC trace of P(**TCT**), obtained with NBu_4_SAc at 50 °C after 180 min (Table S4, Entry 4); integrated as one peak, M_n_,_GPC_ = 16.5 kg/mol, M_w_,_GPC_ = 19.3 kg/mol, Đ = 1.17.

## Spectra – depolymerization

Figure S132: ^1^H-NMR spectra (CDCl_3_) of solid-state ring-closing depolymerization at 155 °C at different time points (Table S5), polythioester (green) and thiolactone (orange).

Figure S133: ^1^H-NMR spectra (CDCl_3_) of optimization base catalyzed ring-closing depolymerization (Table S6) from entry 1 (bottom) to entry 9 (top) polythioester (green) and thiolactone (orange).

Figure S134: ^1^H-NMR spectra (CDCl_3_) of DBU catalyzed ring-closing depolymerization over time (Table S7); polythioester (green) and thiolactone (orange).

Figure S135: ^1^H-NMR spectra (D_2_O) of hydrolysis of P(**TCT**) with 4M NaOH over time (Table S8); TCT-H_2_O (orange) and internal standard (gray).

Figure S136: ^1^H-NMR spectra (D_2_O) of hydrolysis of P(**TCL**) with 4M NaOH over time (Table S9); **TCL**-**H_2_O** (orange) and internal standard (gray).

Figure S137: ^1^H-NMR spectra (MeOD/CDCl_3_) of P(**TCT**) methanolysis with 5 mol% K_2_CO_3_ over time (Table S11); P(**TCT**) in green, **TCT** in orange and **TCT-MeOH** in yellow.

Figure S138: ^1^H-NMR spectra (CDCl_3_/MeOD) of methanolysis of P(**TCL**) with 5 mol% K_2_CO_3_ over time (Table S12); P(**TCL**) in green, **TCL-MeOH** in orange.

# References

[1]	Cioc, R.C. *et al.* (2023). Efficient synthesis of fully renewable, furfural-derived building blocks via formal Diels-Alder cycloaddition of atypical addends. *Green Chemistry*. https://doi.org/10.1039/d3gc02357e.

[2]	Paul, M.K. *et al.* (2024). General and Mild Method for the Synthesis of Polythioesters from Lactone Feedstocks. *ACS Macro Letters*. https://doi.org/10.1021/ACSMACROLETT.4C00556/ASSET/IMAGES/LARGE/MZ4C00556_0002.JPEG.

[3]	Fulmer, G.R. *et al.* (2010). NMR chemical shifts of trace impurities: Common laboratory solvents, organics, and gases in deuterated solvents relevant to the organometallic chemist. *Organometallics*. https://doi.org/10.1021/om100106e.

[4]	Schreurs, A.M.M. *et al.* (2010). EVAL15: A diffraction data integration method based on ab initio predicted profiles. *Journal of Applied Crystallography*. https://doi.org/10.1107/S0021889809043234.

[5]	Sevvana, M. *et al.* (2019). Non-merohedral twinning: From minerals to proteins Sevvana Madhumati. *Acta Crystallographica Section D: Structural Biology*. https://doi.org/10.1107/S2059798319010179/RR5182ZRTI_5_12SUP10.HKL.

[6]	Sheldrick, G.M. (2015). SHELXT - Integrated space-group and crystal-structure determination. *Acta Crystallographica*. https://doi.org/10.1107/S2053273314026370.

[7]	Sheldrick, G.M. (2015). Crystal structure refinement with SHELXL. *Acta Crystallographica*. https://doi.org/10.1107/S2053229614024218.

[8]	Spek, A.L. (2009). Structure validation in chemical crystallography. *Acta Crystallographica*. https://doi.org/10.1107/S090744490804362X.

[9]	Frisch, M.J. *et al.* (2016). *Gaussian 16, Revision C.02*, Gaussian, Inc., Wallingford CT.

[10]	Grimme, S. *et al.* (2010). A consistent and accurate ab initio parametrization of density functional dispersion correction (DFT-D) for the 94 elements H-Pu. *Journal of Chemical Physics*. https://doi.org/10.1063/1.3382344.

[11]	Tomasi, J. *et al.* (2005). Quantum mechanical continuum solvation models. *Chemical Reviews*. https://doi.org/10.1021/CR9904009/ASSET/IMAGES/MEDIUM/CR9904009E00095.GIF.
